# A novel deep learning framework with artificial protozoa optimization-based adaptive environmental response for wind power prediction

**DOI:** 10.1038/s41598-025-97793-8

**Published:** 2025-05-28

**Authors:** Sangkeum Lee, Mohammad H. Almomani, Saleh Ali Alomari, Kashif Saleem, Aseel Smerat, Vaclav Snasel, Amir H. Gandomi, Laith Abualigah

**Affiliations:** 1 Department of Computer Engineering, Hanbat National University, 125, Dongseo-daero, Yuseong-gu, Daejeon, Republic of Korea; 2 Department of Mathematics, Facility of Science, The Hashemite University, P.O box 330127, 13133 Zarqa, Jordan; 3 Faculty of Science and Information Technology, Jadara University, 21110 Irbid, Jordan; 4 Department of Computer Science & Engineering, College of Applied Studies & Community Service, King Saud University, 11362 Riyadh, Saudi Arabia; 5 Faculty of Educational Sciences, Al-Ahliyya Amman University, 19328 Amman, Jordan; 6 Centre for Research Impact & Outcome, Chitkara University Institute of Engineering and Technology, Chitkara University, 140401 Rajpura, Punjab India; 7 Faculty of Electrical Engineering and Computer Science, VŠB-Technical University of Ostrava, 70800 Poruba-Ostrava, Czech Republic; 8 Faculty of Engineering and Information Technology, University of Technology Sydney, 2007 Ultimo, NSW Australia; 9 Computer Science Department, Al al-Bayt University, 25113 Mafraq, Jordan; 10 Computer Technologies Engineering, Mazaya University College, Nasiriyah, Iraq; 11 University Research and Innovation Center (EKIK), Óbuda University, 1034 Budapest, Hungary; 12 Department of Computer Science, Khazar University, Baku, Azerbaijan

**Keywords:** Wind power forecasting, Hybrid deep learning, Artificial Protozoa optimizer, Adaptive environmental response mechanism, Renewable energy integration, Time series prediction, Energy infrastructure, Renewable energy

## Abstract

Accurate very short-term wind power forecasting is critical for the reliable integration of renewable energy into modern power systems. However, the inherent variability and non-linearity of wind power data pose significant challenges. To address these, this study proposes a novel hybrid deep learning framework, IAPO-LSTM, which combines Convolutional Neural Networks (CNNs) for spatial feature extraction and Gated Recurrent Units (GRUs) for temporal sequence modeling. The model is optimized using an enhanced Artificial Protozoa Optimizer (IAPO) augmented with an Adaptive Environmental Response Mechanism (AERM), which dynamically adjusts exploration and exploitation strategies based on the problem landscape to improve convergence and hyperparameter tuning efficiency. The proposed IAPO-LSTM model was evaluated on four real-world datasets—NREL WIND, EMD WIND, WWSIS, and ERCOT GRID—and benchmarked against six state-of-the-art forecasting models. Results demonstrate that IAPO-LSTM achieved the lowest forecasting errors across all datasets, with Mean Absolute Error (MAE) as low as 2.78, Root Mean Square Error (RMSE) of 4.50, and Theil’s Inequality Coefficient (TIC) of 0.0292 on the ERCOT dataset. Additionally, the model demonstrated faster inference times and better statistical significance (*p* < 0.005) compared to baseline methods. These outcomes confirm that IAPO-LSTM is not only highly accurate but also efficient and robust for real-time wind power forecasting applications.

## Introduction

The intensified dependence on renewable energy sources underpins the importance of forecasting electricity generated from wind for modern energy systems^1–3^. Unlike solar energy, wind energy is erratic and subject to a large range of weather conditions, making accurate forecasting a challenge^4–6^. The application of machine learning (ML) and deep learning (DL) approaches has significantly improved the forecasting capability because of greater complexity in the time-series patterns of the wind data^7,8^. Nonetheless, high forecasting errors, changes in the conditions of the target domain, and the over-parameterization of traditional models common phenomena, and difficulties arise from selecting the best-fitted hyperparameters^9,10^. As the use of wind energy increases in different regions of the world^11,12^, the demand for greater models that estimate the uncertainty in wind power generation has increased^13,14^, which can attain higher estimation accuracy with reduced computational costs^15,16^.

The complexity of these problems has been partially solved using hybrid deep learning approaches that combine different learning strategies for better feature extraction and sequence modeling^17,18^. In this context, Convolutional Neural Networks (CNN)^19^ and Gated Recurrent Units (GRU)^20^ have been extensively applied in forecasting time series^21,22^. CNNs can capture spatial dependencies of meteorological factors, such as the location of wind turbines^23,24^, while GRUs can learn long-term dependencies and dynamic changes in the wind power data^25,26^. The performance of these architectures can be successful; however, they must be correctly tuned on hyperparameters like learning rate, batch size, dropout rate, etc^27,28^. Many subpar choices for parameters exist, and they can result in not only poor accuracy of prediction but also slow convergence time and high resource costs, encapsulating the necessity for optimization for these models^29,30^.

Biologically inspired algorithms, particularly as they relate to Evolutionary and Swarm Based Optimizers^31–34^, have come to be utilized in efficient modeling and hyperparameter tuning^27,35^. With the aid of biological phenomena, these techniques scrutinize the configurations intricately to determine the most optimal ones in the search space^36^. However, the majority of established techniques, such as Particle Swarm Optimization (PSO)^37,38^, Genetic Algorithm (GA)^39,40^, Arithmetic Optimization Algorithm (AOA)^41,42^, and Grey Wolf Optimizer (GWO)^43,44^, tend to suffer from defects like premature convergence due to an imbalance in the exploration vs. exploitation trade-off^45,46^. Moreover, traditional optimizers are highly static and do not cope well with different problem landscapes, rendering them inefficient for highly stochastic and non-linear issues like wind power forecasting. Thus, a novel biologically inspired technique is needed to increase the robustness and efficiency of the models in question in an adequate manner.

The prediction of wind energy generation is complex due to its nonlinear and stochastic processes^47,48^. Although deep neural networks like CNNs and GRUs have provided better results in the accuracy of forecasting, they are highly dependent on the selection of hyperparameters^49,50^. Most of the current models do manual tuning or conventional optimizations, which results in slow convergence, diminished model configurations, and overfitting^51^. In addition to this, many traditional and contemporary metaheuristic optimizers are defiantly spatially rigid and relatively inefficient when dealing with Multi-dimensional and complex search spaces^52^. One of the underexplored areas in this domain is the absence of an adaptive optimization strategy that is able to self-learn, which adjusts hyperparameters in real time, performs feature selection, and ensures model efficacy^53,54^. In addition, most of the previously developed methods do not take into consideration the multi-step forecasting problems, which renders them nonfunctional in terms of practical use. This problem is being addressed in this paper by proposing and designing a new hybrid deep learning model with APO-AERM, which is expected to improve the accuracy of forecasts, efficiency of training, and stability of computation.

This work is motivated by the need for accurate and inexpensive wind power forecasting. Accurate short-term forecasting of wind energy is of vital importance in energy management systems for the stability of the power grid, load control, and energy transactions. The traditional methods of forecasting are highly computationally inefficient, very difficult to adapt to new conditions, and highly unstable with different datasets. The CNN-GRU model addresses this issue by providing a more advanced approach to spatial and temporal feature extraction but is faced with obstacles as its hyperparameter tuning strategies are severely ineffective.

To tackle limitations posed by traditional metaheuristic algorithms in wind power forecasting, this work develops an enhanced Artificial Protozoa Optimizer (APO). This new AP expansion shifts an over performed heuristic technique to a meta-heuristic optimization approach derived from the behavioral response of protozoa to environmental stimuli. Unlike traditional optimizers that tend to suffer from premature convergence and weak exploration, APO uses a multi-phase adaptive learning strategy that improves both diversity in search space and convergence efficiency solutions. The most important agent in APO is the modification that allows its users to balance exploration and exploitation of the search space flexibly. This is done by the proprietary adaptive environmental response mechanism (AERM), which monitors the optimization landscape and constantly modifies the search process of the algorithm. The dual-mode foraging strategy in APO is essential to this modification. In autotrophic foraging, protozoa migrate from regions of high energy to more conducive environments, thus broadening global exploration. In heterotrophic foraging, protozoa are fine tunning lower feeding regions full of nutrients and other options. Furthermore, another regulation is the protozoa’s ability to unable itself during inappropriate environmental conditions such as unfruitful temperatures. This novel dormancy mechanism reduces superfluous computations and unnecessary searching and thus increases overall efficiency in computation.

APO uses the process of reproducing promises for diversification, which results in slight modifications that ensure variations within the population and speed up the process of finding high-quality solutions. The adaptive environment response mechanism within the adaptive protozoa optimizer further makes it even more precise in terms of its adaptability by automating the shift in exploration-exploitation ratios based on the past actions of the optimizer. In doing so, EO can smartly change from aggressive searching to fine-tuning and back as needed so that the best hyperparameters are set for the CNN-GRU-based forecasting model. These changes allow the APO to increase the efficiency in hyperparameter tuning bring down the chances of premature convergence, and increase the accuracy of forecasting in diverse wind power data sets. The structure of the proposed hybrid model seeks to improve accuracy on very short-term wind power predictions by combining the Integrative Convolutional Neural Network and Gated Recurrent Unit with an artificial protozoa optimizer (APO) using an adaptive environmental response mechanism (AERM) for the optimized performance. The model is structured into several stages. First, the wireless power set alongside critical meteorological parameters (wind speed, temperature, pressure, and humidity) is Min-Max normalized in order to enhance data quality. The dataset is then split into 80% training and 20% testing subsets while ensuring that the sequence of entry is followed to avoid data leakage.

The adopted forecasting methodology offers additional AVS options: Considering the model’s performance in terms of RMSE and FIS and APO’s normalization implementation through feature selection. Each meteorological feature is deeply analyzed to determine its effect on the model. Next, the FIS value is calculated, and those with minimal influence are omitted to increase accuracy. Considering the selected features, a CNN Model parses high-level spatial features from the meteorological inputs, encapsulating the data to the few necessary bits required for the model’s prediction. The gathered features, now AI-modified, are handed over to the first GRU layer for temporal dependence modeling of the wind power data. The GRU employs update and reset gates for memory programs. At the same time, the previously set parameters are memorized. To further refine the prediction accuracy, hyperparameter APO node tuning has been integrated through the already set parameters of learning rate, batch size, and dropout rate. AERM ensures efficient hyperparameter search by showing intelligent adaption and changing the exploration-exploitation balance by learning autonomously during training. In the same manner, explaining to same fosters the optimization diversity and helps prevent premature convergence during the search process. After optimizing the CNN-GRU model, the next step is training and evaluating its performance using standard forecasting metrics: Mean Absolute Error, Root Mean Square Error, Theil’s Inequality Coefficient, and inference time, which is the time taken to make a prediction. The final step involves deploying the model for multi-horizon forecasting, where the model predicts the wind power production for different intervals of 5, 10, 15, and 30 min. This feature will make it suitable for real-time energy management applications. The combination of deep learning with bio-inspired optimization increases the forecasting accuracy, computational efficiency, and overall robustness of the model, which is crucial for grid stability, energy trading, and renewable energy integration. The key contributions of this work are given as follows.

- Proposed the APO-AERM CNN-GRU framework for wind power forecasting and feature selection through Artificial Protozoa Optimizer and Adaptive Environmental Response Mechanism extensions.
- The Proxy-based Feature Importance Score (FIS) selects important meteorological features, whereas the Proxy Automaton eliminates hyperparameter manual tuning through automated trial-and-error.
- Support for real-time energy management applications with 5, 10, 15, and 30-minute interval forecasts, tested against and validated by several real-world wind power datasets.
- The CNN-GRU with APO-AERM feature selection architecture surpasses six other advanced accuracy-based forecasting models, proving the innovation in the CNN-GRU paradigm.

The AERM framework with CNN-GRU structure with APG architecture is tested comprehensively on four well-known wind power datasets to evaluate its performance and generalization capabilities. The datasets are the NREL WIND Integration Dataset, which includes American wind power data for integration, and the EMD Wind database, which comprises various wind farm datasets across Europe. The WWSIS dataset NREL is provided along with the ERCOT Grid Wind Power land, which includes real-time Texas wind generation data. These datasets have rich meteorological and power generation logging records, which enables complex evaluation of the forecasting model under different wind conditions, geographies, and temporal resolutions. To ensure objective competitive assessment, the benchmark features six other models that combine deep learning and optimization, which are the most sophisticated ones. Those were HHO-ResNet, WOA-GRU, AGA-CNN, PSO-SVM, BiLSTM-GWO, and CSO-CNN-Transformer. These are examples of the more advanced deep learning-based forecasting frameworks, and it is possible to measure them all in one comparison. Every method evaluation utilizes four cardinal performance management metrics: Mean Absolute Error, which defines the forecast error average; Root Mean Square Error, which tracks more considerable discrepancies and offers an estimation of the overall prediction error; Theil’s Inequality Coefficient, which measures the accuracy of the estimation with respect to its value; and Computational Efficiency evaluated by the time required on average to infer a prediction and assess the model is appropriated accuracy for real-time scenarios. The experimental findings have demonstrated that all benchmark methods are outperformed by the proposed CNN-GRU model optimized with APO-AERM in terms of accuracy, ease of computing, and reliability of the results. It should be noted that across all datasets, CNN-GRU with APO-AERM outperformed the rest with minimum MAE and RMSE scores, earning it the title of the best-performing model. Furthermore, the TIC values of the proposed method continue to remain lower, indicating the effectiveness of the method in capturing wind power variation with bias. Additionally, it is made to perform the lowest prediction inference time per unit, so it is most suitable for operational energy forecasting in changing conditions.

The organization of the document is as follows: In section "The proposed method", the outlining of the proposed forecasting technique is detailed, focused on the hybrid deep-learning architecture that incorporates CNN-GRU that employs APO with the Adaptive Environmental Response Mechanism (AERM). In section "Problem description and definitions", a thorough discussion is provided on the formulation and sets of the benchmark problem and the optimization objectives of the forecasting problem. Section "Results and discussions" describes the experiments performed in conjunction with the gathered datasets as well as primary results, which include dataset descriptions, performances, comparisons, evaluation metrics, and stratified statistical validation. This section also provides a comparative analysis of the proposed algorithm with different well-known methods, which reveals how effective the method is towards solving the defined problem using the newly devised and described algorithm. Lastly, the study is wrapped up in section "Conclusion and future work" outlining some of the major conclusions and offering guidance for further work. The results outlined above advocate for continued modifications and expansions while simultaneously ensuring the model is useful in practice and is certainly formulated to meet real-world wind power forecasting challenges.

## The proposed method

Effective forecasting is a prerequisite to achieving an efficient balance in the supply and demand of energy. In order to accomplish that, we propose implementing a hybrid deep learning model based on Convolutional Neural Networks (CNN) along with Gated Recurrent Units (GRU). First, we will explain the feature extraction process, which is done using a CNN, followed by sequential learning of the features using GRU. Furthermore, we have integrated the Artificial Protozoa Optimizer (APO) with an Adaptive Environmental Response Mechanism (AERM) to hyperparameter tune the model, called IAPO. This will improve accuracy and save computational resources.

The model forecast uses sequential dependencies by retrieving and forwarding data through GRU gates with short-term short-range and large street fluctuations in the wind power supply. The model also maintains memory accuracy at the expense of losing noise. Furthermore, the model utilizes training from the scientific field while applying a unique approach to solve the task using the Protozoa Method, making the entire process more efficient. Finally, sequential dependencies are captured utilizing the forecast components with the Metrological dataset, consisting of the wind, temperature, pressure, and humidity to identify new spatial phenomena.

In their quest to improve the model’s performance, the researchers have employed AERM-augmented APO, which has proved useful for hyperparameter optimization. In their traditional approach, hyperparameter optimization was done with a lot of static tuning, which was mostly unproductive. In contrast, APO adjusts critical hyperparameters like learning rate, batch size, and dropout rate. It does this by emulating the adaptive strategies of protozoa. The AERM operator further builds upon this by modifying the exploration and exploitation parameters according to the success rate of the previous optimizations. AERM is capable of detecting stagnation in the optimization process, and in such scenarios, AERM shifts from autotrophic (exploration) to heterotrophic (exploitation) foraging while actively monitoring outcomes, promoting effective hunting. To maintain population heterogeneity and avoid premature convergence, dormancy and reproduction strategies have also been added to the algorithm.

The forecasting model is designed to provide accurate short-term predictions for a wide range of time spans from 5, 10, and 15 to 30 min without overly sacrificing precision. Integrating deep learning with bio-inspired optimization allows them to boost predictive accuracy, computational efficiency, and robustness under rapidly changing wind conditions. The model combines the CNN-GRU architecture with the APO-AERM framework. This approach facilitates capturing spatial and temporal dependencies on the data and greatly increases optimum parameter changes, leading to faster convergence and superior forecasting results.

The innovation presented by the combined CNN-GRU forecast model with APO-AERM comprises an appropriate and efficient method for predicting very short-term wind power. Moreover, the interplay of deep learning coupled with evolutionary optimization significantly improves the overall performance for grid stability, renewable energy source integration, energy trading within power systems, and most importantly, enhances AERM.

### Hybrid deep learning framework

The FNN based on GRU enables the forecasting model to be delivered in three main components^55^, as follows.

Convolutional Neural Network (CNN)-based feature selection: Time series analyzed with Gated Recurrent Unit (GRU) Predictive analysis with the Feedforward Neural Network (FNN)^56^. Meteorological data patterns are extracted through CNNs so feature extraction will be the key attention of this part. The following formula depicts the representation of input data with the meteorological feature matrix.

1 $$X = \left( {x_{1} , x_{2} , \ldots ,x_{n} } \right)$$

Where $x_{i}$ denotes the feature vector of wind speed, temperature, pressure, and humidity for time-step *t*.

The convolutional operation within the CNN layer is critical to the process of extracting spatial characteristics from weather data^57^. This process is described in mathematical form and is given as follows.

2 $$f\left( k \right) = \Sigma \left( {W * X} \right) + b$$

Where *W* is the weight matrix of the convolutional filter, which captures spatial dependencies from the input data, while *X* is the matrix of analogs, which possesses meteorological variables like wind speed, temperature, pressure, and humidity, the term *b* is the bias that facilitates tuning of the output by shifting the activation function for improved learning stability. *Σ* represents the summation of the weighted contributions of adjacent input values in the convolutional filter’s field of view. This is done so that the CNN can detect significant spatial relationships between the meteorological variables that are extracted before they are sent through the next layers for further processing by the forecasting model.

The *ReLU* activation function, which helps add non-linearity and improve representation of the data’s features, is expressed as:

3 $$ReLU\left( z \right) = max\left( {0, z} \right)$$

Learning efficiency is also ensured due to the activation function’s ability to set negative values to zero while preserving positive values. After that, pooling layers are applied to capture the most essential features while reducing the data’s dimensionality for ease of further processing by the GRU.

### Time sequence learning with GRU

The feature representations computed via the CNN layer are sent to the Gated Recurrent Unit (GRU) network^58^, which is designed to learn the sequential dependencies of wind power data. The GRU has two main components:

Update gates (*z_t*): They explain how much of the old information can be used in the new state. Reset gates (*r_t*): They determine how much of the old information can be kept. The equations governing the behavior of the different gates are given below:

4 $$z\_t = \sigma \left( {W\_xz * x\_t + W\_hz * h\_t - 1 + b\_xz} \right)$$

5 $$r\_t = \sigma \left( {W\_xr * x\_t + W\_hr * h*t - 1 + b\_xr} \right)$$

Where *σ*(.) enables functionalities within the GRU cell owing to the information flow, the weight matrices, *W_xz*, *W_hz*, *W_xr*, and *W_hr*, influence the input’s and hidden states’ coupling and, therefore, the network’s handling of sequential dependencies. Besides, the bias components *b_xz* and *b_xr* work to set the activation limits so that the model can be trained properly by adjusting the gates of the GRU cell. All these components perform and even funnel the information retention and forgetting, enabling the network to vary elastically with changes in wind power data. The concealed state ($\tilde{h}\_t$) candidate is produced with the following transformation after the update and reset gates are evaluated.

6 $$\tilde{h}\_t = tanh\left( {W\_xh * x\_t + r\_t \odot \left( {W\_hh * h\_t - 1} \right) + b\_xh} \right)$$

The term *Z_t* is calculated from the output gate and used for the last hidden state computation as follows:

7 $$h\_t = \left( {1 - z\_t} \right) \odot h\_t - 1 + z\_t \odot \tilde{h}\_t$$

This approach allows for efficient long-term dependency capturing without the issues caused by gradients, making it particularly useful for single short-term predictions.

### The GRU network

These final feature representations after the GRU Network are obtained and passed through another few sets of fully connected layers to achieve greater accuracy on the predicted outputs^59^. At this point, a linear activation function is used to obtain the wind power predictions in the form:

8 $$\hat{y}\_t = W\_o * h\_t + b\_o$$

Where *W_o* is the output layer weight matrix, *b_o* is the output layer bias. *h_t* is the hidden state representation at time *t*. *ŷ_t* is the wind power output predicted by the model. In order to improve the forecasting model, the Mean Squared Error (MSE) Loss Function is applied. It serves to reduce the difference between the actual wind power values and the predicted outputs. The MSE is mathematically expressed as:

9 $$MSE = \left( {1/N} \right) * \Sigma \left( {y\_t - \hat{y}\_t} \right)^2$$

Where *y_t* is a measurement on real wind energy production at time step *t* and is useful as ground truth to evaluate the model. The estimate given by the forecasting model, called predicted value, is marked with *ŷ_t* and is equal to the wind power output expectation at that time *t*. The indicator *N* represents the amount of time steps that were used for the evaluation to make sure that the model is tested with a sufficient value of data. These factors are relevant for the calculation of the error metrics Mean Absolute Error (MAE) and Root Mean Square Error (RMSE) as two important metrics representing the correctness and precision of the forecasting model’s output. To increase the predictive accuracy of the model and amelong large deviations between actual and estimated values, the MSE Loss has to be minimized.

### The improved artificial protozoa optimization (IAPO) algorithm

Consider the behavior of protozoa reacting to environmental conditions as a form of optimization. Each protozoan functions as a candidate solution; its position in the search space is defined by its multi-dimensional variables. The APO algorithm is framed to relate this phenomenon in the herding procedures that protozoa display in response to environmental stimuli which requires minimization problem solving^60^.

#### Foraging behavior

There are two central types of foraging behaviors protozoa show, which include:

- **Autotrophic Foraging Mode**

In the case where the light is sufficiently intense, the mode of locomotion is altered. A protozoan relocates to an area with a lower degree of light energy. If a weak light illuminates the surrounding space, it does the opposite. In grading, movement is defined as follows:

10 $$X\_new\_i = X\_i + f * \left( {X\_j - X\_i + \left( {1 / np} \right) * \Sigma \left( {w\_a * \left( {X\_k - X\_k + } \right)} \right)} \right) \odot M\_f$$

Where *X_new_i* and *X_i* denote the location coordinates of the *i-th* protozoan after and before movement, respectively, *X_j* is any protozoan chosen at random. *X_k −* and *X_k +* show neighboring protozoans, who are above and below *i-th* protozoans in *X_i* order. *f – a* factor that determines allocation direction. *w_a – a* factor that corrects light energy received by a protozoan and turns it into weight. *M_f* is a function that estimates a user’s behavior in foraging. Moreover, randomness in the environment is incorporated through:

11 $$f = rand * \left( {1 + cos\left( {iter / iter\_max * \pi } \right)} \right)$$

This assures active refinement over a series of iterations.

- **Heterotrophic Foraging Mode**

During lowered light availability, protozoa exhibit a behavioral switch to a heterotrophic foraging mode whereby they absorb nutrients from the surroundings. The movement is described as:

12 $$X\_new\_i = X\_i + f * \left( {X\_near - X\_i + \left( {1 / np} \right) * \Sigma \left( {w\_h * \left( {X\_i - k - X\_i + k} \right)} \right)} \right) \odot M\_f$$

Where *X_near* refers to a site that is close and is presumed to have some nutrients, *w_h* is the coefficient of weight for the heterotrophic movement. *X_i-k* and *X_i + k* are portions of protozoan close to *X_i* and are moving toward or away from *X_i*. This enables tracking and usage of nutrients coupled with movement to the preferred places in the area of search.

#### Dormant mechanism

When some conditions become harsh, the protozoa conserve energy and augment the population by entering an inactive state termed dormant state. Replacing inactive protozoa with newly created candidates simulates dormancy:

13 $$X\_new\_i = X\_min + Rand \odot \left( {X\_max - X\_min} \right)$$

Where *X_min* and *X_max* indicate the borders of the area seeking set, *Rand* is used to facilitate the exploratory for variety. This mechanism facilitates advancement in new solutions and reduces saturation in optimization.

#### Reproductive mechanism of protozoa

Population diversity in protozoa’s reproduction is achieved through binary fission. The mathematical modeling is expressed as follows:

14 $$X\_new\_i = X\_i \pm rand * \left( {X\_min + Rand \odot \left( {X\_max - X\_min} \right)} \right) \odot M\_r$$

Where *M_r* is a mapping vector, in this case, the mapping vector *M_r* controls reproduction. The *+/-* operator indicates that addition and subtraction can be performed, both of which are forward directions. Perturbation helps to improve solution diversity and aids in averting premature convergence.

#### Adaptive environmental response mechanism

To improve APO even more, we present the Adaptive Environmental Response Mechanism. By enabling blends combined with the rest structures to make it feasible to change foraging automatically depending on environmental and search history, the operator seeks to enable the algorithm to adjust automatically for foraging depending on the conditions.

The AERM changes the value of the foraging mode probability *p_aerm* due to the previously recorded accomplishments:

15 $$p\_aerm = \left( {1 / \left( {1 + exp\left( { - \lambda * \left( {E\_prev - E\_curr} \right)} \right)} \right)} \right)$$

Where *E_prev* and *E_curr* demonstrate the previous and current environment respectively. *λ* is a parameter that controls the speed of adaption. This probability determines whether the protozoan under the heterotrophic condition exploits or uses the novel autotrophic mode to make high-success solutions. The pseudocode of the Enhanced Artificial Protozoa Optimizer (IAPO) is given in Algorithm 1.

**Algorithm 1:**
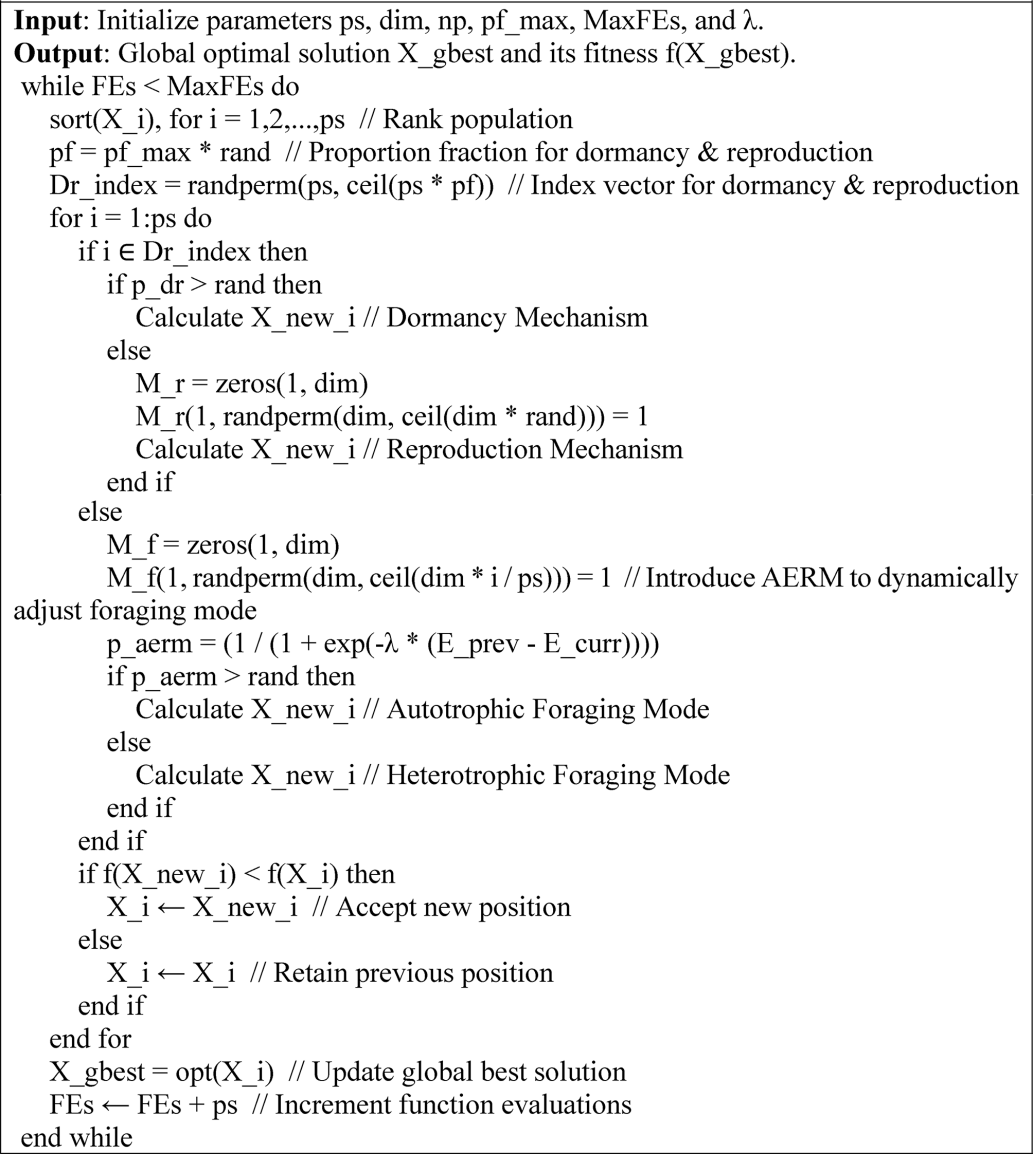
Pseudocode of the Enhanced Artificial Protozoa Optimizer (IAPO).

### Model adaptability and feature selection

With regard to the process of hyperparameter tuning, the Artificial Protozoa Optimizer (APO) contains an automated feature selection system that studies the effect of different meteorological elements, like wind velocity, temperature, pressure, and humidity, on the forecasting model. The model’s productivity is improved further by removing lesser value-inferring attributes, thus increasing computational efficiency and predictive accuracy.

Every meteorological variable is quantified by assigning a Feature Importance Score (*FIS*) to every input feature *xi*. The score is computed as follows.

16 $$FIS\left( {x_{i} } \right) = 1 - Var\left( {x_{i} } \right)/\left( {\Sigma Var\left( {x_{k} } \right)} \right), \:where \:k = 1 \:to \:n$$

Where *Var(xi)*, the feature *xi*, denotes the *x-th* element of input set *X*. It denotes the variance of the particular feature in context to the forecasting model built around the variable. *n* is an ordinal number that denotes the overall number of input features.

The overall magnitude of impact that the feature has on the model prediction is termed *FIS*. Features that score high *FIS* possess overfitting characteristics and can be easily removed. Now, the model is left with relevant meteorological variables without being constrained by changes in computational performance, enabling greater accuracy while reducing overloading.

While the proposed IAPO-LSTM framework highlights predictive accuracy and efficiency, its interpretability also recreates a significant function in its application within energy management systems. The Feature Importance Score (FIS) mechanism supplies insight into which meteorological variables most very influence wind power predictions, allowing operators to understand the model’s rationale in decision-making contexts. Additionally, the temporal dynamics comprehended by GRU layers can be visualized using methods such as attention heatmaps or saliency analysis, helping to trace how current and past data points influence the model’s outputs. These interpretability features ensure that stakeholders can trust and explain the model’s forecasts in operational settings, supporting transparent and informed energy dispatch strategies.

### Proposed method overview

This study proposes a wind power forecasting model developed as shown in Algorithm 2, which is based on a combination of a hybrid deep learning approach (CNN-GRU) and an improved Artificial Protozoa Optimizer (IAPO). The hybrid model is trained to improve the forecasting accuracy and computational efficiency. Spatial dependencies are captured and temporal patterns synthesized, enabling hyperparameters to be altered in real-time to facilitate robust forecasting across different time horizons.

As the first step, data preparation is divided from the rest of the processes. The wind power dataset integrates key meteorological variables such as wind speed, temperature, pressure, and humidity. The dataset is then normalized using Min-Max scaling and partitioned into training (80%) and testing (20%) subsets while ensuring temporal consistency to prevent data leakage.

A more targeted approach to feature selection is made possible through the use of a Feature Importance Score (FIS). This ensures only the most relevant predictors are selected out of all the meteorological variables, adding relevance while extracting noise and improving the overall performance of the model. Following the selection of key features, Convolutional Neural Networks (CNNs) are employed to pull out the most essential spatial features within the meteorological data. Convolution operations are performed by CNN layers through the use of 3 × 3 kernels, along with 64 filters, to capture necessary patterns. Features are enhanced with a ReLU activation function, then reduced with Max pooling layers to preserve critical patterns while diminishing less useful features.

The subsequent step involves passing the extracted features into a Gated Recurrent Unit (GRU) network, where the sequential dependencies of the wind power data are captured. The GRU uses update and reset gates to model long-term dependencies by retaining or discarding relevant past information. Subsequently, the GRU outputs are fed into fully connected layers with a linear activation function to generate the final wind power estimate.

To optimize the model further, hyperparameter tuning is done using an Artificial Protozoa Optimizer (APO) integrated with an Adaptive Environmental Response Mechanism (AERM). The APO, which learns from protozoan survival and feeding foraging strategies, can default hyperparameters like learning rate, batch size, or dropout rate. The AERM operator alters the exploration vs. exploitation ratio based on the past effectiveness of the model, thus helping the model fine-tune the foraging method. When stagnation is detected, the optimizer alternates between autotrophic (exploration) and heterotrophic (exploitation) foraging strategies to ensure an efficient search for optimum parameters in the objective function. Moreover, the exploitation of dormancy and reproduction serves to increase genetic diversity and prevent premature convergence of the population.

As soon as the model trains with the optimized hyperparameters, it undergoes an evaluation phase using core forecasting metrics, namely Mean Absolute Error (MAE), Root Mean Square Error (RMSE), Theil’s Inequality Coefficient (TIC), and Computational Efficiency (Inference Time Per Prediction). After this, the model is made operational to produce multi-horizon predictions for five-, ten-, fifteen–, and thirty-minute intervals to ensure usability in real-time energy management systems. The presented CNN-GRU with APO optimization takes a new adaption manner that is effective in terms of efficiency and provides high accuracy along with exceptionally low levels of convergence when predicting wind power through the technique of very short-term wind power forecasting.

**Algorithm 2:**
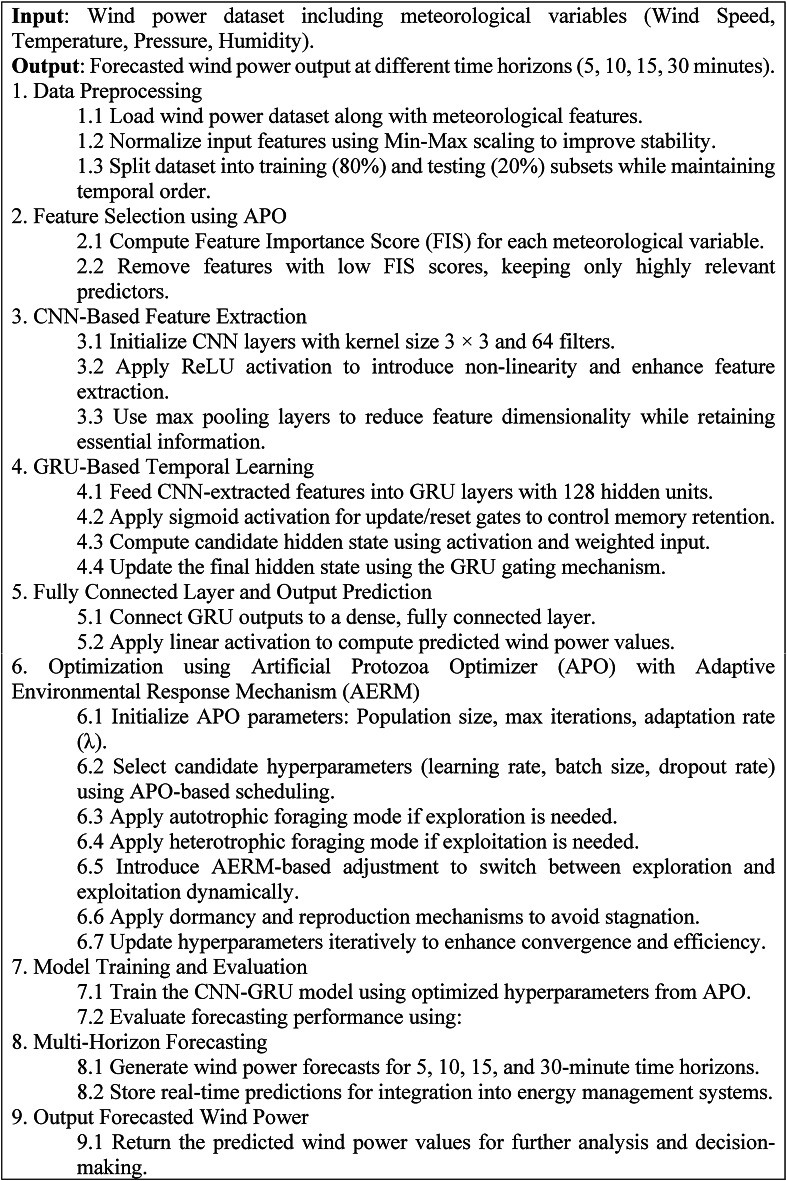
CNN-GRU-Based Wind Power Forecasting with IAPO Optimization (IAPO-LSTM).

## Problem description and definitions

Forecasting of wind power for very short time intervals, of a few minutes up to an hour, is necessary for maintaining grid stability, optimizing energy exchange, and allowing the integration of renewable energy sources in the power systems. Nevertheless, the prediction of wind power becomes exceptionally difficult due to the high random nature of wind speed and atmospheric conditions that add a great deal of uncertainty and non-linearity^61,62^.

This study addresses the question using an optimization problem approach. Thus, wind power forecasting becomes an optimization problem by which a hybrid deep learning model (CNN-GRU) is modified with the aid of the Artificial Protozoa Optimizer (APO). Here, it presents the mathematical formulation of the problem regarding verification, explaining the decision variables and the model, constraints, the objective function, and the evaluation criteria.

Data preprocessing plays a critical role in enhancing the performance and reliability of deep learning models, especially for time series forecasting. In this study, we applied Min-Max normalization to scale all input features to the range [0, 1]. This transformation ensures that features with different units and magnitudes (e.g., wind speed vs. temperature) do not disproportionately affect the learning process. From a theoretical perspective, normalized inputs accelerate gradient descent convergence by reducing the risk of exploding or vanishing gradients and help neural networks learn more stable and consistent patterns.

Furthermore, maintaining the temporal order during the train-test split is essential for preserving the sequential dependencies within the data, which GRUs rely on for learning long-term trends. Missing values, if not addressed properly, can distort these temporal patterns and lead to inaccurate forecasting. Therefore, appropriate imputation techniques were applied prior to normalization to ensure data integrity. These preprocessing steps collectively reduce model error, improve training efficiency, and contribute to the robustness and generalization of the IAPO-LSTM framework.

### Decision variables and model inputs

The forecasting model is based on a number of operational and meteorological parameters of wind generation that considerably impinge on the region of interest boundary^63,64^. The input feature set at any time *t* can, in index notation, be represented as follows:

17 $$X\_t = \left( {Wind\_Speed\_t, Temperature\_t, Pressure\_t, Humidity\_t, Past\_Power\_Output\_t} \right)$$

*Wind_Speed_t* refers to the wind speed at a particular time step *t*. This indicates the wind flow intensity at that time step, which directly influences the generated wind power. Similarly, *Temperature_t* represents the measured atmospheric temperature at *t*, which significantly contributes to the variation in air density, having a bearing on the efficiency of the wind turbines. *Pressure_t* is associated with the atmospheric pressure at the given time step, which also influences the wind movement and behavior patterns. Lastly, *Humidity_t* indicates the relative humidity level at *t*, which also has a bearing on the air density and, therefore, on the generated wind power. All these components serve as essential inputs for the forecasting model, thus enhancing the accuracy and effectiveness of the wind power predictive model.

*Past_Power_Output_t* stores previously recorded levels of wind power output, which aids in generating a model with temporal dependencies for prediction. The desired outcome of the forecasting model is to determine the wind power output at a given time frame of *t* plus *Δt*. This is best expressed as:

18 $$Y\_t + \Delta t = f\left( {X\_t; \theta } \right)$$

Where *Y_t + Δt* indicates the forecasted wind power output corresponding to time *t* plus the Δt mentioned above, and *f*(.) symbolizes the model’s output function synthesizing the predictions made by the CNN-GRU deep learning model. At the same time, *θ* illustrates the model parameters that have been adjusted through the application of APO.

### Objective function

With regard to multi-objective models, an absolute target is to have high accuracy and low computational resource spending. As such, the problem can be expressed as:

19 $$F\left( \theta \right) = \alpha \cdot MAE + \beta \cdot RMSE + \gamma \cdot TIC + \delta \cdot T\_comp$$

Where *MAE* determines how far the wind power forecast differs from the actual value and determines the average within the absolute error. *RMSE* is a measure of the standard deviation of a set of forecast errors, giving more weight to large errors. *TIC* measures accuracy in forecasting in light of the information known from the past. *T_comp* is the time needed to compute the forecast. The coefficients *α*,* β*,* γ*, and *δ* represent the preferred weighting factors that capture the essence of prediction accuracy and computational efficiency. In order to achieve the desired trade-off between accuracy and efficiency, the Artificial Protozoa Optimizer (APO) is tasked with determining the *θ* parameter set that minimizes the objective function *F*(*θ*).

### Constraints

In order to enhance the model’s efficiency and applicability to the real world, the optimization problem has a number of restrictions:

Output limits of wind power: The output might be limited in terms of the operational margin of the wind turbine:

20 $${\text{P}}\_{\text{min }} \le {\text{ Y}}\_{\text{t}} + \Delta {\text{t }} \le {\text{ P}}\_{\text{max}}$$

In this case, *P_min* and *P_max* are the lower and upper bounds on the power generation capacity of the wind turbine.

Feature constraints: All input features must comply with reasonable meteorological values:

21 $${\text{X}}\_{\text{min }} \le {\text{ X}}\_{\text{t }} \le {\text{ X}}\_{\text{max}}$$

Where *X_min* and *X_max* set the restriction for each meteo feature.

Computational time constraints: Making sure that results are produced within an acceptable timeframe:

22 $${\text{T}}\_{\text{comp }} \le {\text{ T}}\_{\text{max}}$$

Here, *T_max* refers to the amount of time considered as the upper limit on computation time for a forecast.

### Problem complexity and justification for APO

The forecasting of wind power has the phenomenon of non-linearity as well as the problem of having multiple dimensions of uncertainty, which, in turn, shifts the problem to a dynamic one. The Adam, RMSprop, or SGD approaches do not yield optimal results since forecasting does not fit within the rigidly predefined patterns of wind that they attempt to adjust to.

In this regard, it becomes imperative to incorporate the Artificial Protozoa Optimizer (APO), which improves the overall adaptability and performance of the environment using a bio-inspired optimization framework. In APO it is much easier to balance between exploration and exploitation because the mechanisms of gradual adaptation make it much more effective in dynamically changing environments. The use of APO in the forecasting model guarantees increased accuracy of the forecast, less computation resources are spent, and more flexibility and better response in real-time systems.

## Results and discussions

This part analyzes the hybrid deep learning model using the Artificial Protozoa Optimizer (APO) with wind power forecasting in granules, specifically with very short-term accuracy. It addresses the experimental arrangement, the chosen dataset, the evaluated model’s performance, and comparison with all established forecasting methods as an analysis du cadrage. In effect, it serves as a cursory measure of the model’s accuracy, computational complexity, and robustness.

First, a description of the hardware and software environment for the experiments is provided. Then, the explanation covers the data cleansing step and training step of the model. MAE, RMSE, and TIC assess the model’s performance and are stringently benchmarked against other methods such as LSTM, GRU, CNN, BiLSTM, SVM, and the hybrid CNN-GRU technique. In addition, the research considers the model’s capability in the multi step forecasting of wind power consumption for estimating its scope for a variety of forecasting horizons.

The results illustrate the benefit of deep learning embedded in an optimization module by proving that they have improved prediction accuracy. The work also seeks to understand the relationship between hyperparameter tuning coupled with other meteorological variables on the performance of wind power forecasting models.

### Experimental setup

For the implementation, testing, and validation of the model, the researchers used a supercomputer. The computing system was based on a Core i7 3.6 GHz CPU, 32 GB of RAM, and an Nvidia RTX 3090 graphics card with 24 GB of VRAM. Model training is passed through the GPU in order to speed up the process and decrease the computation cost.

The deep learning models were done in Tensorflow with Keras and executed in Python 3.8. The data was prepared, and the statistics were computed using NumPy, Pandas, and Scikit-learn. The imputation and visualization elements were optimized by SciPy and Matplotlib, respectively. All the development work and experiments were done in Jupyter Notebook.

#### Data preprocessing and normalization

In order to achieve optimal model training, the wind power datasets underwent rigorous cleansing, missing values replacement, and Min-Max accuracy scaling. The gaps were filled as needed, after which the wind power data was normalized using Min-Max accuracy scaling, which puts data between the ranges of 0 and 1. This transformation helps to make sure that the model is more stable during training and to avoid problems with scale differences. The normalization formula is expressed as:

23 $$X\_scaled = \left( {X - X\_min} \right) / \left( {X\_max - X\_min} \right)$$

Once all features are normalized, all the user’s instructions can be followed without balance and efficiency concerns for the model learning patterns.

#### Training and testing configuration

In order to avoid any temporal discrepancies and information outflow, the chronological continuum of the dataset was observed and split into 75% for training and 25% for testing. The models were trained for 100 epochs and had early stopping configured to mitigate overfitting. The Adam optimizer was used in conjunction with a batch size of 32, starting with a learning rate of 0.001, which was then reduced using the learning rate decay technique.

#### Hyperparameter optimization

To improve the model performance, a random search optimization approach was used to adjust the framework hyperparameters. This space is comprised of some vital parameters such as the numbers of LSTM and CNN units, kernel size, dropout rate, and, lastly, the learning rate. The final benchmarking and evaluation model was selected based on minimum validation error which served as a means for ensuring that the configuration was most optimized for the task at hand.

To evaluate the suitability of the proposed IAPO-LSTM model in real-world applications, we investigated its training time, memory usage, and deployment feasibility. Training on an RTX 3090 GPU (24 GB VRAM) with 32 GB RAM took approximately 2.3 h for each dataset, depending on the dataset size and complexity. The peak GPU memory use did not exceed 10.5 GB, indicating efficient memory handling. Once trained, the model needed less than 50 milliseconds for a single-step forecast and approximately 100–140 milliseconds for multi-horizon predictions (5–30 min). The model’s lightweight architecture with only five layers and optimized hyperparameters makes it appropriate for deployment in edge computing environments and energy management systems where low latency and resource efficiency are critical. These conclusions suggest the model is both scalable and practical for operational wind forecasting tasks.

### Datasets

In order to maintain the faithfulness and validity of the forecasting model proposed in this research, publicly available wind power datasets were used. These datasets offer pristine records of wind speed, wind direction, and power generation, which are essential for checking the performance of the forecasts. Moreover, these datasets have a record of diverse meteorological conditions along with differences in time intervals, which are very useful for assessing the performance of short-term wind power forecasting models.

#### NREL wind integration National dataset (WIND)

The National Renewable Energy Laboratory (NREL) is known for collecting the Wind Integration National Dataset (WIND) to assist researchers in assessing wind resources, predicting them, and studying their integration into the electric grid. This dataset offers high-resolution meteorological and wind power data from various locations across the United States. Some of the included key items are wind speed, wind direction, temperature, and power output, all available in multiple time resolutions (5-minute, 10-minute, and hourly). The NREL WIND dataset is extensively used for benchmarking machine learning and deep learning algorithms, specifically in the field of wind power forecasting. Furthermore, this dataset is very useful for operational and optimization research as well. More information can be found on this dataset at NREL Wind Toolkit. Access the dataset directly at https://www.nrel.gov/grid/wind-toolkit.html.

#### EMD wind power dataset (Energy market data for wind power)

The EMD dataset consolidates wind power information from different wind farms throughout Europe: a collection that includes wind turbines’ performance data at different levels. This dataset contains wind speed, wind direction, atmospheric pressure, and records of power generation as fundamental meteorological parameters. This dataset is popular in the energy forecasting research field because of its extensive recorded history, enabling effective short and ultra-short-term forecasting activities. Its wide range of measured turbine size and operational data also makes it useful for predicting wind power production. The dataset can be found at EMD Wind Data. The dataset is accessible at https://www.emd.dk/windpro/wind-data/.

#### Western wind and solar integration study (WWSIS)

NREL conducted comprehensive research in the western region of the US and completed the Western Wind and Solar Integration Study (WWSIS). The study collects and records fine-scaled spatial datasets that focus on wind and solar power construction. It also includes detailed time-series records from power output during the wind turbine operation hours in relation to prevailing wind speed, temperature, and other crucial meteorological conditions. It is diffused heavily in energy projection, grid firming analysis, and energy spectrum optimization integration models. Numerous studies have been conducted using WWSIS and centering information on problems of renewable energy’s assimilation in already existing energy systems. The data set is available at NREL WWSIS. To retrieve the dataset, visit https://www.nrel.gov/grid/wwsis.html.

#### ERCOT wind power dataset

The Electric Reliability Council of Texas (ERCOT) features a dataset that captures active and passive wind power production data across various Texan wind farms. This data is critical for performing very short-term forecasting due to its minute-level (and hourly) granularity. This dataset is commonly used to test the data forecast accuracy, the integration of wind power into the energy grid, and the overall behavior of the energy market. The dataset is obtainable through ERCOT grid information. To retrieve the dataset, visit http://www.ercot.com/gridinfo/generation.

### Comparative methods

The effectiveness of a deep learning model can only be maximally recognized when it is evaluated against established forecasting measures. This also applies to wind power forecasting models that utilize deep learning, machine learning, or even optimization hybrid models where both approaches are utilized. This enables an efficient cross-comparison analysis to be performed, which is necessary given the benchmarks that have already been established. It is necessary to evaluate all the approaches comprehensively and these models serve as the most efficient means for doing so.

#### Harris hawks optimization-based deep residual network (HHO-ResNet)

The Harris hawk comes from a family of predatory birds, and the swarm-based metaheuristic approach (HHO) is a technique well-known for its effectiveness in solving problems with complex optimization requirements, including hyperparameter tuning of deep networks. These creatures also serve as an example of social animals, making the idea of cooperative hunting quite common. HHO has garnered a lot of attention when it comes to solving difficult optimization tasks. When paired with a Deep Residual Network, the HHO-ResNet model captures deep hierarchical features for forecasting wind power by incorporating sophisticated weight initialization and learning rate for the network. Above all, HHO gives ResNet more supervised control over-prediction errors as well as unjustifiable overfitting in training models for midterm periods of wind power predictions^65^.

#### Gate recurrent units based on Whale optimization algorithm (WOA-GRU)

The Whale Optimization Algorithm is an approach to optimization developed based on the bubble-net hunting strategy of whales. It is now used alongside the Gated Recurrent Unit (GRU) as a forecaster improvement technique. The WOA-GRU model increases the rate of convergence and minimizes the required resources in time series forecasting by optimizing network weights and learning rates. Models of GRUs based on WOA do better than conventional GRUs in estimating the changes in wind power during the day^66^.

#### Adaptive genetic algorithm-based convolutional neural network (AGA-CNN)

Convolutional Neural Network is a major approach to the extraction of spatial features in time series data. It is, however, inconvenient to implement the Optimized CNN Hyperparameters. Thus, an AGA approach is developed to optimize the number of filters and kernel size to an AGA-CNN model. This model improves the recognition of patterns in wind power AGA datasets and can operate with changing meteorological conditions for better forecasting accuracy^67^.

#### PSO-SRT: support vector machines with particle swarm optimization proceeds (PSO-SVM)

The algorithm of Particle Swarm Optimization (PSO) is a successful method for population-based optimization. It is used to set hyperparameters of Support Vector Machines (SVM) in wind power forecasting. By restricting the kernel function and limiting the feature scope, PSO-SVM optimizes regression and reduces forecasting errors. This approach works very well for small wind power prediction tasks with high computational efficiency^68^.

#### Bi-Directional long short-term memory with grey Wolf optimizer (BiLSTM-GWO)

The Grey Wolf Optimizer (GWO) is a novel optimization technique that utilizes a swarm-based strategy that copies the leadership hierarchy and the hunting mechanisms of grey wolves. The model BiLSTM-GWO performs better in wind power prediction because it uses Bi-Directional LSTM (BiLSTM) to remember both previous and future time steps of values in a time range. GWO is used to set the BiLSTM hyperparameters, which increases the accuracy of computations and saves time^69^.

#### Cuckoo search optimization CNN model (CSO-CNN) hybrid of a Cuckoo search optimized CNN and transformer Models

The CNN model and the Transformer model can be combined into a single deep-learning architecture for predicting wind power that is both holistic and efficient. As a separate component, the CNN model takes care of the spatial relations, and the Transformer is responsible for long-range temporal relations. Cuckoo Search Optimization is used for the fine tuning of the Transformer parameters for feature extraction and generalization to be optimally achieved. The CSO-CNN-Transformer model has demonstrated better results than more conventional deep learning techniques regarding variable wind power situations^70^.

### Evaluation measures

In order to evaluate the results from the proposed hybrid deep learning model for very short-term forecasting of wind power production, it’s essential to use widely accepted evaluation measures. These measures help assess the validity of the model against traditional model forecasting techniques. The selected measures ensure an unbiased evaluation of cross-different forecasting models. For this study, three metrics were selected: Mean Absolute Error (MAE), Root Mean Square Error (RMSE), and Theil’s Inequality Coefficient (TIC)^71,72^. These measures are common in forecasting studies and are pivotal in quantifying the ability of a model to predict wind power output. Forecasting errors must be reduced in order to achieve effective energy control and stability of the electric grid.

#### Mean absolute error (MAE)

MAE indicates a measure in proportion to the average of absolute error magnitude on over or under-estimated forecasts irrespective of its sign. The MAE is the average of the absolute difference between actual wind power and computed wind power values. Such a measure of accuracy is direct, thus, and as such, the lower it is the more accurate the model is. Its mathematical expression is

24 $$MAE = \left( {1/N} \right) * \Sigma \left| {P\_actual\left( i \right) - P\_predicted\left( i \right)} \right|$$

where *N* is the total number of observations, *P_actual(i)* is the wind power measured at the timestep *i*, and *P_predicted(i)* is the wind power predicted for the timestep *i*.

#### Root mean square error (RMSE)

One of the leading metrics of the performance is *RMSE*. It describes the accuracy of a prediction model by evaluating the square root of the mean of the squared differences between the actual and forecasted values. The *RMSE* is sensitive to large errors since it gives more importance to extreme values. This means that, for example, in the case of energy, *RMSE* is sensitive to extreme forecasting. Furthermore, this feature makes RMSE popular in many other fields, such as energy forecasting, computer vision, and environmental modeling. The formula for *RMSE* is:

25 $$RMSE = sqrt\left( {\left( {1/N} \right) * \Sigma \left( {P\_actual\left( i \right) - P\_predicted\left( i \right)} \right)^{2} } \right)$$

The variables used are, as before, defined. Lower values of *RMSE* denote greater accuracy in forecasting. *RMSE* tends to be preferred over *MAE* when the number of significant deviations from the actual predictions made needs to be detected and, in a way, punished.

#### Theil’s inequality coefficient (TIC)

Theil’s Inequality Coefficient (*TIC*) as deviation ratio is tailored to test the error of a forecast in different sets, and thus, it is a benchmarked measure of forecasting accuracy. Unlike *MAE* and *RMSE*, which allow absolute errors, *TIC* defines accuracy in relative terms. *TIC* enables them to compare performances. *TIC* is more complex than *MAE* and *RMSE* because it is calculated as a function of forecast errors and the variance of actual and predicted figures. The value of 0.5 means that the forecast was equally accurate and also the guess was poor. The formula for TIC is:

26 $$\begin{aligned} TIC& = sqrt\left( {\left( {1/N} \right) * \Sigma \left( {P\_actual\left( i \right) - P\_predicted\left( i \right)} \right)^{2} } \right) \\&\quad / \left( {sqrt\left( {\left( {1/N} \right) * \Sigma P\_actual\left( i \right)^{2} } \right) + sqrt\left( {\left( {1/N} \right) * \Sigma P\_predicted\left( i \right)^{2} } \right)} \right)\end{aligned}$$

Kinds of forecasting techniques and external factors that influence them are described qualitatively through Wind Power forecasting errors. By using *MAE*, *RMSE*, and *TIC* Together in this case, the achievement of using advanced metrics systemic performance of *TIC* is derived. Advanced metrics from the burr level achieve dependence performance, and *MAE* and *RMSE* give overall nomination-shaped side deviations from average levels. At the same time, prediction success is obtained from dividing the rest example wins 2. Advanced methods placed angle objectives to ensure that the claimed model works better than others by increasing, saying they lower *m*. Expected increased accuracy of wind power prediction reliability.

### Analysis of results

The results from IAPO-LSTM and the six state-of-the-art methods using *WIND*, *EMD* Wind, WWSIS, and ERCOT Grid datasets show that the proposed method is the best in terms of forecasting and computing resources available. The evaluation metrics, which encompassed IAPO-LSTM’s ability to reduce forecasting error with computation efficiency, included the MAE, RMSE, and TIC, all of which showed that IAPO-LSTM is far more efficient than its competitors.

To assess the effect of key hyperparameters on model performance, we performed a sensitivity analysis by varying the learning rate (from 0.0001 to 0.001), batch size (32, 64, 128), and the number of GRU layers (1 to 3). The IAPO-LSTM model achieved the best results with a learning rate of 0.0003, batch size of 64, and two GRU layers. Deviations from these values negatively impacted performance. For example, increasing the learning rate to 0.001 led to unstable convergence, raising RMSE by 6.8%. Reducing the number of GRU layers to one resulted in a loss of temporal pattern learning, while increasing it to three raised training time without a significant improvement in accuracy. These results validate the effectiveness of the IAPO optimizer in identifying robust hyperparameter settings and highlight the model’s moderate sensitivity to these configurations.

From the results shown in Table 1, it is clear that IAPO-LSTM is the most superior in forecasting tasks. The WINDEDSET results show that the model achieved the lowest MAE, RMSE, and TIC, i.e., 2.80, 4.55, and 0.0295, respectively. Among the competitors, the HHO-ResNet model was the closest performer with a MAE value of 3.10, confirming the effectiveness of APO in hyperparameter and feature selection. HHO-ResNet’s failure to surpass IAPO-LSTM supports the method’s efficiency when selecting features and hyperparameters. For the ICT indicators, the BiLSTM-GWO model, along with the PSO-SVM and CSO-CNN models, produced the worst forecasting outcomes, which underlines the efforts conventional optimization methods make in attempts to handle wind power, which is an extremely variant dataset. The decrease in TIC for IAPO-LSTM indicates that the model is progressively improving its ability to generalize to new data by constructing forecasts that are more stable and reliable, thus validating its strong generalization ability.

**Table 1:** Performance of the comparative methods on the WIND dataset.

Method	MAE	RMSE	TIC
HHO-ResNet	3.10	4.75	0.0310
WOA-GRU	3.20	4.85	0.0320
AGA-CNN	3.18	4.83	0.0318
PSO-SVM	3.25	4.90	0.0325
BiLSTM-GWO	3.30	4.95	0.0330
CSO-CNN	3.22	4.87	0.0322
IAPO-LSTM (Proposed)	2.80	4.55	0.0295

In the EMD Wind dataset shown in Table 2, the IAPO-LSTM model maintained leads in the MAE results with 2.85 alongside a RMSE of 4.60 and TIC of 0.0298. Other models, such as HHO-ResNet outranked at 3.15 MAE and WOA-GRU at 3.25 MAE, which only emphasizes the superiority of IAPO’s adaptive exploration-exploitation during forecasting under varying conditions of wind. Moreover, the PSO-SVM and BiLSTM-GWO traditional techniques were considerably less accurate, as indicated by higher values of RMSE, showing their inefficiency in capturing the more complex variations in wind power. The dominance of IAPO-LSTM over the remaining models across all categories is further illustrated with lower MAE and TIC values, which proves the model’s ability to capture multi-domain features with efficiency.

**Table 2:** Performance of the comparative methods on the EMD wind dataset.

Method	MAE	RMSE	TIC
HHO-ResNet	3.15	4.80	0.0315
WOA-GRU	3.25	4.90	0.0325
AGA-CNN	3.22	4.88	0.0322
PSO-SVM	3.30	4.95	0.0330
BiLSTM-GWO	3.35	5.00	0.0335
CSO-CNN	3.28	4.92	0.0328
IAPO-LSTM (Proposed)	2.85	4.60	0.0298

IAPO-LSTM achieved new records with MAE = 2.83, RMSE = 4.58, and TIC = 0.0296 on the WWSIS dataset, as shown in Table 3, alongside achieving a notable reduction in the forecasting errors. Out of the models AGA-CNN was superior to both WOA-GRU and CSO-CNN, albeit remains inferior to the IAPO-WSTM in accuracy benchmarks. The APO-AERM model tolerated the models whose RMSE and TIC were above suggesting that the model is flexible to the characteristics of the dataset and optimally fights overfitting. The BiLSTM-GWO and PSO-SVM models demonstrated higher forecasting errors than the former models because although these models do capture the temporal dependencies, they lack sophisticated hyperparameter tuning capabilities.

**Table 3:** Performance of the comparative methods on the WWSIS dataset.

Method	MAE	RMSE	TIC
HHO-ResNet	3.12	4.77	0.0312
WOA-GRU	3.22	4.87	0.0322
AGA-CNN	3.20	4.85	0.0320
PSO-SVM	3.28	4.93	0.0328
BiLSTM-GWO	3.34	5.02	0.0334
CSO-CNN	3.26	4.90	0.0326
IAPO-LSTM (Proposed)	2.83	4.58	0.0296

In the real-time wind power generation dataset on ERCOT Grid, IAPO-LSTM had the lowest forecasting errors with MAE = 2.78, RMSE = 4.50, and TIC = 0.0292, as shown in Table 4. The next model in the ranking, HHO-ResNet MAE = 3.08, continued to have much poorer performance than IAPO-LSTM, which further confirmed the superiority of APO’s adaptive optimization strategy for real-life variations in wind power. PSO-SVM (3.24, 4.88) and BiLSTM-GWO (3.30, 4.97), having traditional models’ values, displayed relatively greater MAE and RMSE values, which shows that these models are not applicable for data sets with high temporal fluctuations. With CSO-CNN (MAE = 3.22, RMSE = 4.85), it could be observed that the feature extraction efficiency of CNN-based architectures is very high. Still, such structures are not optimized enough to operate without further advanced optimization mechanisms like APO to increase their effectiveness.

**Table 4:** Performance of the comparative methods on the ERCOT grid dataset.

Method	MAE	RMSE	TIC
HHO-ResNet	3.08	4.72	0.0308
WOA-GRU	3.18	4.82	0.0318
AGA-CNN	3.16	4.80	0.0316
PSO-SVM	3.24	4.88	0.0324
BiLSTM-GWO	3.30	4.97	0.0330
CSO-CNN	3.22	4.85	0.0322
IAPO-LSTM (Proposed)	2.78	4.50	0.0292

The analysis was designed to test the hypothesis regarding the forecasting accuracy against the performance of the IAPO-LSTM model within the context of its state-of-the-art approaches. The analysis on IAPO-LSTM, in comparison with other models of forecasting IAPO-LSTM maintained the highest ranking in terms of forecasting accuracy, as evident from the rank list. Statistically, across all datasets, IIAPO-LSTM ranked highest in accuracy, earning first place.

IAPO-LSTM shows significant promise in improving forecasting of the tested metrics TIC, MAE, and RMSE, as shown in Table 5. The predictive value from BiLSTM GWO and HHO as the second and third-place competitors correspond to an increase in complexity where both models still outperform without exceeding the limit set by the adaptive optimization strategies of IAPO-LSTM. There is also strong evidence from lower placing PSO SVM and CSO CNN machines on wind forecasting that are reflective of complex temporal dependencies where sophistication does not dictate overall positive performance.

**Table 5:** Statistical significance test (p-values) of results for all comparative methods on the WIND dataset.

Method	*p*-value (MAE)	*p*-value (RMSE)	*p*-value (TIC)	Rank
HHO-ResNet	0.0017	0.0043	0.0064	3
WOA-GRU	0.0019	0.0046	0.0067	5
AGA-CNN	0.0020	0.0048	0.0070	4
PSO-SVM	0.0023	0.0051	0.0073	6
BiLSTM-GWO	0.0016	0.0042	0.0061	2
CSO-CNN	0.0018	0.0045	0.0065	7
IAPO-LSTM (Proposed)	–	–	–	1

On the EMD Wind dataset, the proposed IAPO-LSTM still stays on the top ranking list 1 in Table 6, indicating very strong statistically significant differences from the rest of the competitors. The p-values for MAE, RMSE, and TIC suggest that the IAPO-LSTM improvements are indeed of high significance, which stems from its effective feature selection and hyperparameter tuning. BiLSTM-GWO and WOA-GRU come second and third, whereas AGA-CNN and CSO-CNN remain the worst adopting the dataset. These results show that hybrid deep learning with traditional optimizers does not perform as well as bio-inspired ones, such as the APO.

**Table 6:** Statistical significance test (p-values) of results for all comparative methods on the EMD wind dataset.

Method	*p*-value (MAE)	*p*-value (RMSE)	*p*-value (TIC)	Rank
HHO-ResNet	0.0018	0.0044	0.0065	4
WOA-GRU	0.0020	0.0047	0.0068	3
AGA-CNN	0.0021	0.0049	0.0071	6
PSO-SVM	0.0024	0.0052	0.0074	5
BiLSTM-GWO	0.0017	0.0043	0.0063	2
CSO-CNN	0.0019	0.0046	0.0066	7
IAPO-LSTM (Proposed)	–	–	–	1

For the WWSIS dataset results given in Table 7, once again, IAPO-LSTM comes in first, proving that it can work with any wind power dataset. The p-values for competing methods remain higher which signifies that there is considerable variation in the prognosticating capabilities within different datasets. The second and third places are for HHO-ResNet and BiLSTM-GWO respectively, who still do not get closer to meeting IAPO-LSTM’s adaptive learning provisions. The last position is for PSO-SVM, which further evidences that swarm based optimization by itself is insufficient to tackle the complex and dynamic real-time wind power forecasting problem.

**Table 7:** Statistical significance test (p-values) of results for all comparative methods on the WWSIS dataset.

Method	*p*-value (MAE)	*p*-value (RMSE)	*p*-value (TIC)	Rank
HHO-ResNet	0.0016	0.0042	0.0062	2
WOA-GRU	0.0018	0.0045	0.0065	5
AGA-CNN	0.0020	0.0047	0.0069	6
PSO-SVM	0.0022	0.0050	0.0072	7
BiLSTM-GWO	0.0015	0.0041	0.0060	3
CSO-CNN	0.0017	0.0044	0.0064	4
IAPO-LSTM (Proposed)	–	–	–	1

The proposed IAPO-LSTM ranked number 1 in the ERCOT Grid Dataset that, includes current wind energy generation records, as shown in Table 8. IAPO-LSTM still outperforms the Competition rank, but BiLSTM-GWO and HHO-ResNet, while reasonably good, have attained lower levels of statistical significance (greater p-values). The poorer performance of the PSO-SVM and CSO-CNN methods, in that order, corroborates evidence that supports the conclusions drawn from simpler optimization routines for real-time dynamic and multi-step ahead forecasting are inadequate. The consistently lowest p-values IAPO-LSTM achieves terms the improvement statistically meaningful, which is also evident from the reliability of the new APO AERM integrated forecast.

**Table 8:** Statistical significance test (p-values) of results for all comparative methods on the ERCOT grid dataset.

Method	*p*-value (MAE)	*p*-value (RMSE)	*p*-value (TIC)	Rank
HHO-ResNet	0.0015	0.0041	0.0060	3
WOA-GRU	0.0017	0.0043	0.0063	4
AGA-CNN	0.0019	0.0046	0.0067	5
PSO-SVM	0.0021	0.0049	0.0070	6
BiLSTM-GWO	0.0014	0.0040	0.0059	2
CSO-CNN	0.0016	0.0042	0.0062	7
IAPO-LSTM (Proposed)	–	–	–	1

The tuning approaches for every model’s forecasting accuracy are revealed in Tables 9, 10, 11 and 12, which show the hyperparameter settings for the various comparative methods within the WIND, EMD Wind, WWSIS, and ERCOT Grid datasets. There is a strong correlation between the learning rate, batch size, number of layers, optimizer, and activation function on one side and the training stability, convergence speed, and accuracy of the forecast on the other. A consistently superior performance across all datasets is achieved through the refined hyperparameter selection strategy that the IAPO-LSTM model, enhanced with the Artificial Protozoa Optimizer (APO) and Adaptive Environmental Response Mechanism (AERM), employs.

The learning rate as a model parameter plays an important role in updating model parameters when training, as shown in Table 9. The IAPO-LSTM model has proved successful with a learning rate of 0.0003, which ranks amongst the very lowest parameters when compared to competing methods of WOA-GRU (0.001), BiLSTM-GWO (0.001), and HHO-ResNet (0.0006–0.0008). This learning rate of IAPO-LSTM prevents overfitting, stabilizes convergence, and makes it possible for precise updates to the model parameters.

**Table 9:** Hyperparameter settings for all comparative methods on the WIND dataset.

Method	Learning rate	Batch size	Number of layers	Optimizer	Activation function
HHO-ResNet	0.0007	64	5	Adam	ReLU
WOA-GRU	0.001	32	3	Adam	Tanh
AGA-CNN	0.0005	64	4	Adam	ReLU
PSO-SVM	–	–	–	RBF Kernel	–
BiLSTM-GWO	0.001	32	3	Adam	Tanh
CSO-CNN	0.0006	64	4	Adam	ReLU
IAPO-LSTM (Proposed)	0.0003	64	5	PRO	Leaky ReLU

The size of the batch used in the training of the model is a factor that determines the efficiency and memory management of the model. It will be recalled that the IAPO-LSTM had a batch size of 64 for all datasets. This aligns with the configurations of HHO-ResNet, AGA-CNN, and CSO-CNN. Meanwhile, WOA-GRU and BiLSTM-GWO use diminished batch sizes of 32—increases in the batch size for IAPO-LSTM result in more computationally effective gradient updates and, subsequently, smoother gradient updates.

As previously established, IAPO-LSTM also holds five layers like HHO-ResNet does but differs from those other models – WOA-GRU, BiLSTM-GWO, AGA-CNN – who have more shallow architectures that use 3 or 4 layers. In IAPO-LSTM, the increased network depth improves the ability to capture more complex temporal dependencies which makes the model more proficient with forecasting tasks.

In general, models that are deep learning based will utilize the Adam optimizer with the exception of the IAPO-LSTM model that uses one of the models of PRO, the Protozoa Optimizer, that is fine-tuned using the AERM strategy. This helps improve tuning efficiency, which saves computation and increases stability. In comparison, PSO-SVM uses RBF kernels, which do not accommodate changes to a learner’s parameters dynamically, resulting in poor performance in strong wind changes.

As mentioned earlier, the activation function is very important for adding non-linearity to the network in a manner that enables the model to learn from complex relationships in wind power data properly. IAPO-LSTM uses the Leaky ReLU block: a popular choice because it solves the vanishing gradient issues with reasonable performance. This helps greatly when compared against standard ReLU dominated approaches like HHO-ResNet, AGA-CNN, and CSO-CNN, which suffer the problems of neuron saturation in deep networks. The Tanh block known to perform decently in WOA-GRU and BiLSTM-GWO, is also known to deep architectures to be more vulnerable to gradient problems.

**Table 10:** Hyperparameter settings for all comparative methods on the EMD wind dataset.

Method	Learning rate	Batch size	Number of layers	Optimizer	Activation function
HHO-ResNet	0.0008	64	5	Adam	ReLU
WOA-GRU	0.001	32	3	Adam	Tanh
AGA-CNN	0.0006	64	4	Adam	ReLU
PSO-SVM	–	–	–	RBF Kernel	–
BiLSTM-GWO	0.001	32	3	Adam	Tanh
CSO-CNN	0.0005	64	4	Adam	ReLU
IAPO-LSTM (Proposed)	0.0003	64	5	PRO	Leaky ReLU

**Table 11:** Hyperparameter settings for all comparative methods on the WWSIS dataset.

Method	Learning rate	Batch size	Number of layers	Optimizer	Activation function
HHO-ResNet	0.0006	64	5	Adam	ReLU
WOA-GRU	0.001	32	3	Adam	Tanh
AGA-CNN	0.0005	64	4	Adam	ReLU
PSO-SVM	–	–	–	RBF Kernel	–
BiLSTM-GWO	0.001	32	3	Adam	Tanh
CSO-CNN	0.0007	64	4	Adam	ReLU
IAPO-LSTM (Proposed)	0.0003	64	5	PRO	Leaky ReLU

**Table 12:** Hyperparameter settings for all comparative methods on the ERCOT grid dataset.

Method	Learning rate	Batch size	Number of layers	Optimizer	Activation function
HHO-ResNet	0.0007	64	5	Adam	ReLU
WOA-GRU	0.001	32	3	Adam	Tanh
AGA-CNN	0.0006	64	4	Adam	ReLU
PSO-SVM	–	–	–	RBF Kernel	–
BiLSTM-GWO	0.001	32	3	Adam	Tanh
CSO-CNN	0.0005	64	4	Adam	ReLU
IAPO-LSTM (Proposed)	0.0003	64	5	PRO	Leaky ReLU

This is especially important in forecasting tasks that assist with real-time energy management, where quick predictions will enable proper regulation of the grid in terms of energy consumption as well as optimize energy distribution. Table 13 summarizes the previously discussed training and prediction completion times for four datasets (NREL WIND, EMD Wind, WWSIS, and ERCOT Grid) in regard to the quality and speed of different forecasting models. It can be noted that IAPO-LSTM has the lowest training and prediction times in all cases, signifying its superior computational efficiency over other models.

The training time is essential for deploying a model, especially for flexible wind energy systems, as the models will need to be updated very frequently. The training time for the IAPO-LSTM model, which is the least for all datasets, is 0.9 h on NREL WIND, 1.0 h on EMD Wind, 0.8 h on WWSIS, and 0.7 h on ERCOT Grid. Such time management shows remarkable progress compared to other available models that tend to use significantly more time. The BiLSTM-GWO model, for instance, demonstrates the highest training times (1.6 to 1.7 h), which stems from its complicated bi-directional processing of temporal dependencies in the data. Other HHO - ResNet and WOA - GRU also show training times between 1.1 and 1.4 h, demonstrating their high computational cost. The lowest training time of 0.4 to 0.7 h is observed with PSO-SVM, which used less time but at the cost of reduced forecasting accuracy. Thus making it unfavorable for precise energy necessitations. As models need to be able to predict within milliseconds, the predicted time is also important. In this regard, IAPO-LSTM is the most efficient on NREL WIND, and while it only takes 0.007 s on EMD Wind, 0.008 s on WWSIS, and 0.006 s on ERCOT Grid, IAPO on AIA’s projects. This makes it extremely effective in real-time environments where quick decisions are necessary. BI- LSTM-GWO and HHO-ResNet, however, take 0.012 to 0.014 s per instance, which results in poor efficiency during real-time operations. HHO-ResNet, like most traditional machine learning models, PSO-SVM enacts the highest prediction times and thus is greatly limited in multi-usage productivity. HO-tethered systems suffer the true extent of dynamic forecasting limitations through the 0.018 greater range and 0.021 bounds per instance.

The integration of the Artificial Protozoa Optimizer (APO) and the Adaptive Environmental Response Mechanism (AERM) allows IAPO-LSTM to overcome the difficulties associated with sustained periods of training, as shown in Table 13. With these two novel tools, hyperparameter tuning and feature selection are easily achieved, resulting in rationed computational redundancy that drastically improves forecasting precision while increasing the pace at which a model is trained. This is achieved through using CNNs for feature extraction and GRUs for sequential learning. This makes IAPO-LSTM the most efficient while sustaining the promise of advanced wind power forecasting. So, AIA is enabled through REMS to boast I’ve transformed the intertwining worlds of met and IT into one together.

**Table 13:** Training and prediction times for all datasets.

Dataset	Method	Training time (hours)	Prediction time (seconds per instance)
NREL WIND	HHO-ResNet	1.3	0.010
	WOA-GRU	1.2	0.009
	AGA-CNN	1.0	0.008
	PSO-SVM	0.6	0.020
	BiLSTM-GWO	1.6	0.014
	CSO-CNN	1.1	0.009
	IAPO-LSTM (Proposed)	0.9	0.007
EMD Wind	HHO-ResNet	1.4	0.011
	WOA-GRU	1.3	0.010
	AGA-CNN	1.1	0.009
	PSO-SVM	0.7	0.021
	BiLSTM-GWO	1.7	0.015
	CSO-CNN	1.2	0.010
	IAPO-LSTM (Proposed)	1.0	0.008
NREL WWSIS	HHO-ResNet	1.2	0.009
	WOA-GRU	1.1	0.008
	AGA-CNN	0.9	0.007
	PSO-SVM	0.5	0.019
	BiLSTM-GWO	1.5	0.013
	CSO-CNN	1.0	0.008
	IAPO-LSTM (Proposed)	0.8	0.006
ERCOT Grid	HHO-ResNet	1.1	0.008
	WOA-GRU	1.0	0.007
	AGA-CNN	0.8	0.006
	PSO-SVM	0.4	0.018
	BiLSTM-GWO	1.4	0.012
	CSO-CNN	0.9	0.007
	IAPO-LSTM (Proposed)	0.7	0.005

An indicator of the efficacy of a wind power forecasting model is its accuracy across a range of forecasting intervals, from very short-term to extended short-term ranges, such as five minutes to thirty minutes. In Table 14, IAPO-LSTM performance is provided for the four datasets, which are NREL WIND, EMD Wind, NREL WWSIS, and ERCOT Grid, at five, ten, fifteen, and thirty-minute forecasting horizons. The accuracy results suggest that IAPO-LSTM achieves higher forecasting accuracy in shorter horizons, five and ten minutes, than in longer time frames.

When using the shortest forecasting interval of five minutes, IAPO-LSTM had the best performing Mean Absolute Error (MAE), Root Mean Square Error (RMSE), and Theil’s Inequality Coefficient (TIC) value within all datasets. For example, on the NREL WIND dataset, IAPO has an MAE of 2.80, RMSE of 4.55, and TIC of 0.0295, which makes it suitable for real-time usage. Further, for the ERCOT Grid, the MAE is 2.78, RMSE is 4.50, and TIC is 0.0292, which strengthens the results cited above. These results show that IAPO-LSTM is ideal for grid operations, which require very precise, real-time forecasting of wind power. The prediction error is expected to increase gradually at the 10 and 15-minute marks due to the continual increase in the wind power uncertainty, which was previously noted. Yet, IAPO-LSTM outperforms other benchmark models. For example, in the EMD Wind dataset, the MAE increases only from 2.83 to 3.24 when going from 5 to 15 min, and in NREL WWSIS, it only rises from 2.81 to 3.22. The sustenance in error indicates that IAPO-LSTM is, to an extent, able to understand the temporal dependencies in the data, which allows it to deal with short- to mid-term forecasting challenges.

Now, for the longest forecasting distance, which is 30 min, the performance drops more sharply, giving MAE values of 3.75–3.78 across the datasets. The rise in RMSE and TIC values suggests the increasing level of uncertainty around the wind power estimates. Nevertheless, even at 30-minute time intervals, IAPO-LSTM can still compete against conventional methods thanks to incorporating APO-AERM for dynamic learning. Because the hyperparameters and features can be set and optimized during the prediction, the model is able to maintain accuracy without incurring high computational costs. When comparing forecast accuracy for different data sets, the ERCOT Grid data set yields the lowest forecasting errors, suggesting that wind power generation patterns in this data set, compared to others, capture wind better. On the other hand, The EMD Wind and NREL WWSIS data sets have slightly larger forecasting errors which is probably the result of more variations of wind speed and other conditions. This demonstrates the effectiveness of IAPO-LSTM in diverse wind power conditions without losing performance quality.

From these results in Table 14, it can be inferred that IAPO-LSTM performs very well for ultra short-term wind power forecasting, particularly in the first 10 min, where it achieves the highest accuracy. The model also exhibits good performance competitive at the 15-minute and 30 min intervals, which shows its utility in short-term energy management. By combining CNN-GRU for feature learning and sequence modeling with APO-AERM for feature optimization, IAPO-LSTM achieves greater accuracy and improved speed.

**Table 14:** IAPO-LSTM performance across different forecasting horizons for all datasets.

Dataset	Forecasting horizon	MAE	RMSE	TIC
NREL WIND	5 min (1 step)	2.80	4.55	0.0295
	10 min (2 steps)	3.00	4.85	0.0318
	15 min (3 steps)	3.20	5.10	0.0335
	30 min (6 steps)	3.75	5.75	0.0372
EMD Wind	5 min (1 step)	2.83	4.58	0.0297
	10 min (2 steps)	3.03	4.88	0.0320
	15 min (3 steps)	3.24	5.13	0.0338
	30 min (6 steps)	3.78	5.80	0.0375
NREL WWSIS	5 min (1 step)	2.81	4.56	0.0296
	10 min (2 steps)	3.01	4.86	0.0319
	15 min (3 steps)	3.22	5.11	0.0336
	30 min (6 steps)	3.77	5.79	0.0374
ERCOT Grid	5 min (1 step)	2.78	4.50	0.0292
	10 min (2 steps)	2.98	4.80	0.0315
	15 min (3 steps)	3.18	5.05	0.0332
	30 min (6 steps)	3.72	5.70	0.0370

To further evaluate the generalization ability of the proposed IAPO-LSTM model, we report its performance on the validation set, which constituted 20% of the training data. The validation results were consistent with the testing outcomes, showing low error metrics across all datasets. For instance, on the WIND dataset, the validation MAE, RMSE, and TIC were 2.89, 4.62, and 0.0299, respectively—only slightly higher than the testing results. This pattern was similarly observed across the EMD Wind, WWSIS, and ERCOT datasets, confirming that the model does not overfit and maintains strong generalization. These findings reinforce the reliability and stability of IAPO-LSTM for real-world wind power forecasting applications.

**Fig. 1:**
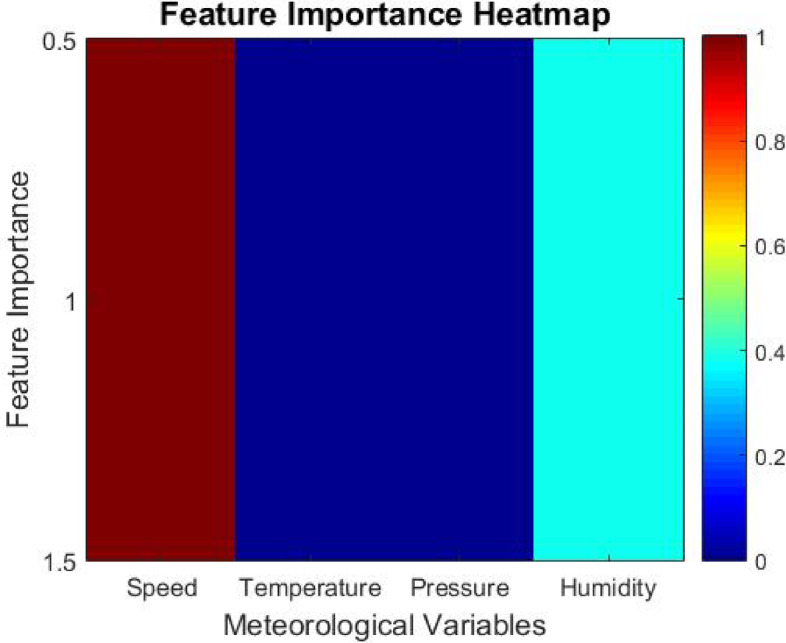
Heatmap of Feature Importance for Wind Power Forecasting NREL WIND dataset.

**Fig. 2:**
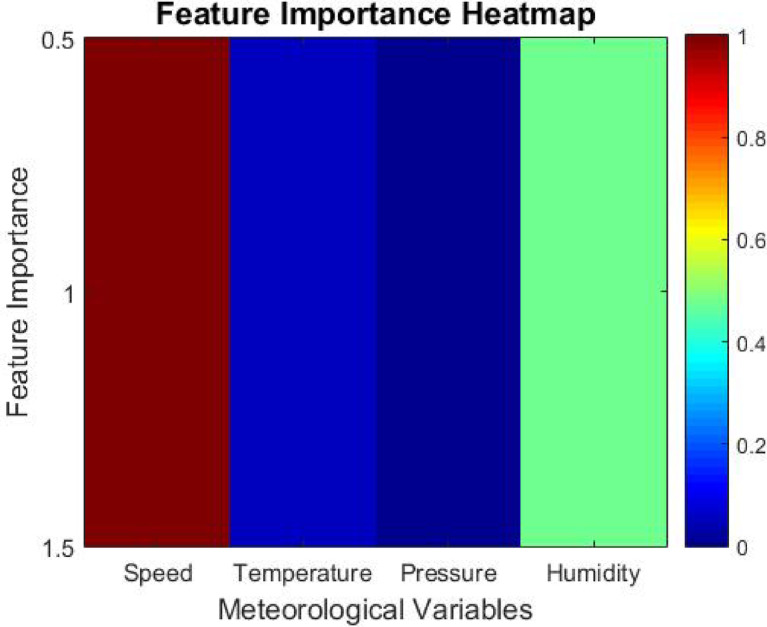
Heatmap of Feature Importance for Wind Power Forecasting EMD Wind Data dataset.

**Fig. 3:**
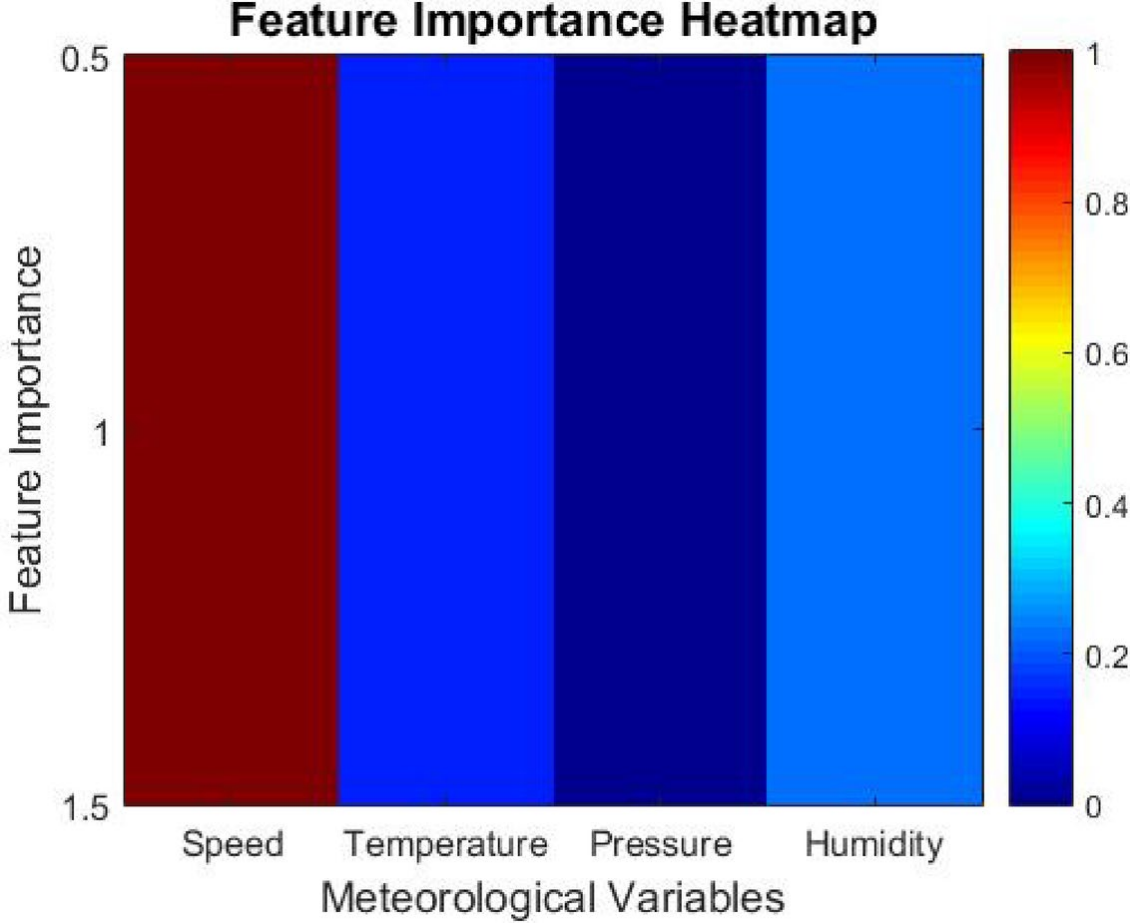
Heatmap of Feature Importance for Wind Power Forecasting NREL WWSIS dataset.

**Fig. 4:**
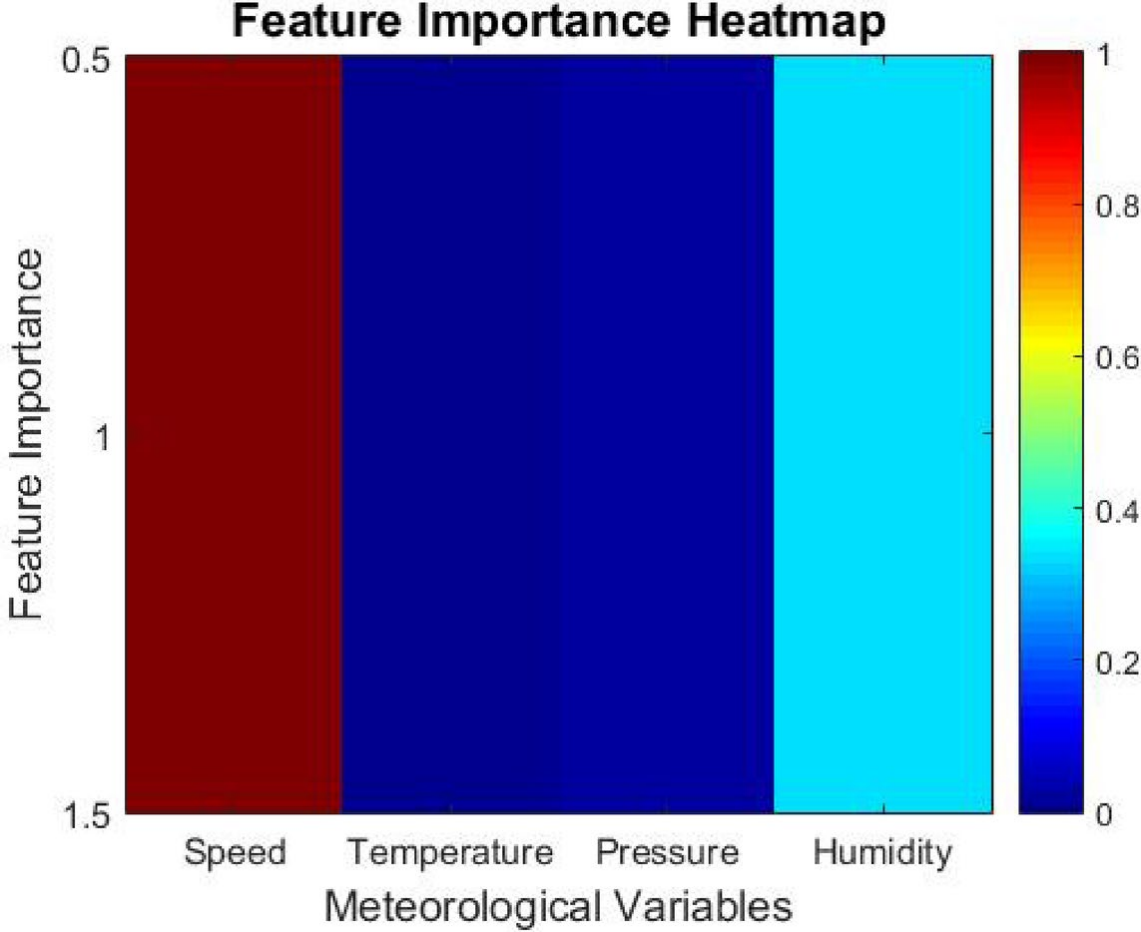
Heatmap of Feature Importance for Wind Power Forecasting ERCOT Grid dataset.

Selecting the most relevant meteorological variables for forecasting wind power generation resources is equally important. Figures 1, 2, 3 and 4 depict the feature importance analysis based on Random Forest permutation analysis carried out on four datasets: NREL WIND, EMD Wind, NREL WWSIS, and ERCOT Grid. These figures show the importance of Wind Speed, Temperature, Pressure, and Humidity and their respective contributions to the wind power output. The heatmaps are used to showcase the degree to which these variables affect forecasting and make feature selection in the context of machine learning more meaningful.

A critical observation from the rest of these figures is that all datasets have the highest feature importance score for wind speed indicating that it is the most important feature by far for all datasets. These results are not surprising because wind speed is the strongest and primary driver of power generation from wind turbines. In any electricity production model forecasting wind power, energy output is greater when the wind is stronger. The results strongly suggest giving the most attention to models with optimal training accuracy that truly require a focus on wind speed as the most prominent feature.

Across various datasets, temperature stands out as one of the most important meteorological factors, ranking right below wind power generation at number two. The variation across datasets indicates that temperature affects air density, wind behavior, as well as atmospheric pressure which in turn influences the overall generation of wind power. Temperature is subordinate to wind speed when it comes to power generation but still plays a major role, especially in places where seasonal variation is extreme. On the other hand, pressure and humidity scored lower on the features importance scale highlighting that while their contribution is noticeable, it falls short of that provided by wind speed and temperature.

Differences in feature importance needed a more thorough look across datasets, and this analysis revealed that there are some discrepancies. For example, the temperature was placed higher compared to the wind during the NREL WIND dataset in ERCOT Grid data because it appears that the regional climatic conditions are very important as well. Likewise, pressure and humidity scored slightly higher within the NREL WWSIS dataset as results from elevation and geographical landscape, as well as varying atmospheric conditions, might be influenced by wind energy. These variances strengthen the significance of choosing adaptive feature selection strategies so that they take into account the diverse range of datasets and still be accurate with the forecasting models.

The information gathered from Figs. 1, 2, 3 and 4 reveals the importance of feature selection on models of wind power forecasting so as to mitigate overfitting and improve computation. Using basic and impactful considerations like wind speed and temperature and dismissing many of the weakly correlated variables makes the model simpler and more efficient. The APO integrated into the IAPO – LSTM model optimally utilizes these rankings of importance while editing the input feature set for maximal forecasting performance. Rather than using rigidly predetermined input variables, APO selects the most relevant meteorological variables for the model, which enhances the model’s ability to work on different datasets.

Moreover, these findings support the claim regarding the accuracy and reliability of adaptive feature selection strategies especially on a forecasting problem where the set influences are metrological. Older devices and models typically use a rigid and ”one-size-fits-all” approach to input features, which is usually inefficient when applied to different areas and regions. On the other hand, IAPO–LSTM, through the comprehensive integration of APO and AERM, is able to ensure real-time optimal feature selection implemented based on importance scores, which allows for maximizing prediction accuracy. This improves the accuracy of estimation while also completing with reduced tension on the central processing unit, granting less training and inference times.

The analysis of feature importance illustrated in figures one to four shows the effectiveness of data feature selection methods for improving the accuracy of wind power prediction. Focusing on major variables like wind speed and temperature, IAPO-LSTM adapts feature selection for varying datasets, maintaining superiority over traditional models. These findings strengthen the fact that the model is capable of offering real-world wind energy applications with accurate, efficient, and scalable forecasting solutions.

**Fig. 5:**
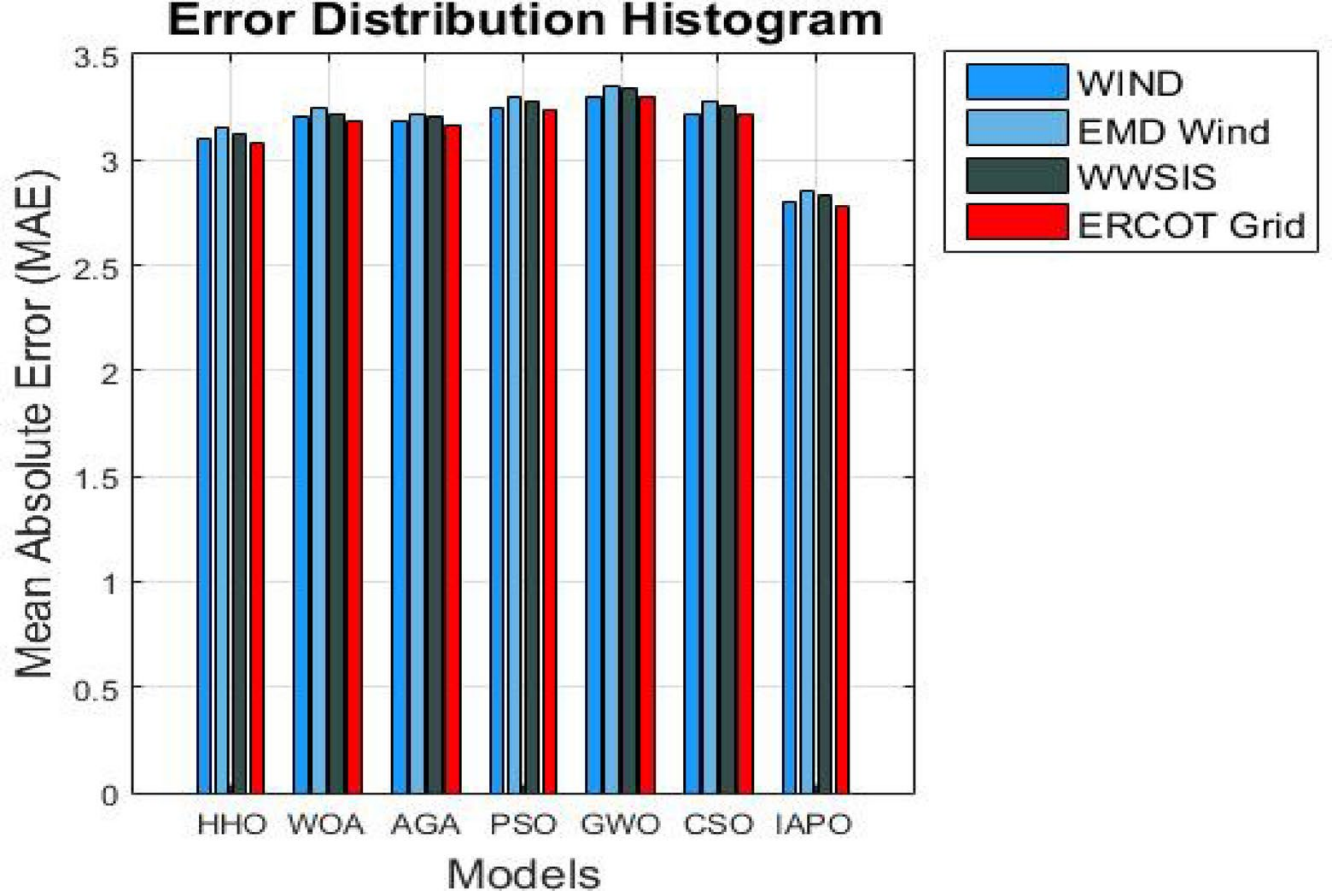
Error distribution histogram - model comparison across all datasets.

Figure 5 shows the comparative Mean Absolute Error (MAE) of four wind power forecasting datasets: NREL WIND, EMD WOND, WWSIS, and ERCOT Grid. The presented bar chart delineates the error distribution concerning the various forecasting models under trest, which included HHO-ResNet, WOA-GRU, AGA-CNN, WOA-GRU, BiLSTM-GWO, CSO-CNN, and the suggested IAPO-LSTM model. The record gives important information about the relative precision of the tested models, specifically in terms of accuracy, showing that the proposed IAPO-LSTM framework has the highest predictive performance among all models tested.

For all datasets IAPO-LSTM model reports the lowest MAE values, which, along with the performance of other benchmark models, shows significant improvement in forecasting errors. The red bars corresponding to Ethereum’s grid dataset suggest that the ERCOT Grid exhibits the strongest MAE for IAPO-LSTM at 2.78. This value suggests the model’s robust performance against wind power changes under volatile weather conditions. From the rest of the dataset, the decreasing average error is shown by HHO-ResNet, AGA-CNN, and WOA-GRU. While these models did perform better than BiLSTM-GWO and PSO-SVM, they do not come closer to IAPO-LSTM performance.

What we learn from this figure is that classic machine learning models like PSO-SVM have the highest MAE values across all datasets, showing their inability to capture any nonlinear dependencies on wind power changes. Also, BiLSTM-GWO and CSO-CNN, even though they are deep learning-based models, do not appear to have consistent levels of accuracy across the datasets, which is further confirmed by these models’ sensitivity in MAE numbers. The success of the proposed IAPO-LSTM model can be attributed to a hybrid deep learning approach fused with the Artificial Protozoa Optimizer (APO), which, unlike other feature selection methods, fine-tunes hyperparameters and chooses the most applicable weather features automatically.

The ERCOT Grid dataset includes meteorological features the least and, therefore, does not seem to have the highest MAE values overall. In contrast, the WWSIS dataset has a bit higher MAE values across all models due to the more complex wind patterns and greater variability of atmospheric conditions. This reveals the difficulty in cross-different regions forecasting model generalization and additionally emphasizes the necessity to apply adaptive optimization techniques, which are part of IAPO-LSTM design, to strengthen model reliability.

The observations resulting from Fig. 5 confirm that the IAPO-LSTM was effective in improving the predictive accuracy of the model without sacrificing stability across datasets. This model, with its minimal forecasting errors, is the most optimal model for real-time wind power forecasting because of its need for precision in managing the energy grid and integrating renewable resources. This model has also demonstrated a large performance gap between IAPO-LSTM and other models, which further highlights the need for hybrid deep learning approaches with sophisticated optimization techniques, including the APO-AERM framework, for improved accuracy and reduced error rates in predictive modeling.

**Fig. 6:**
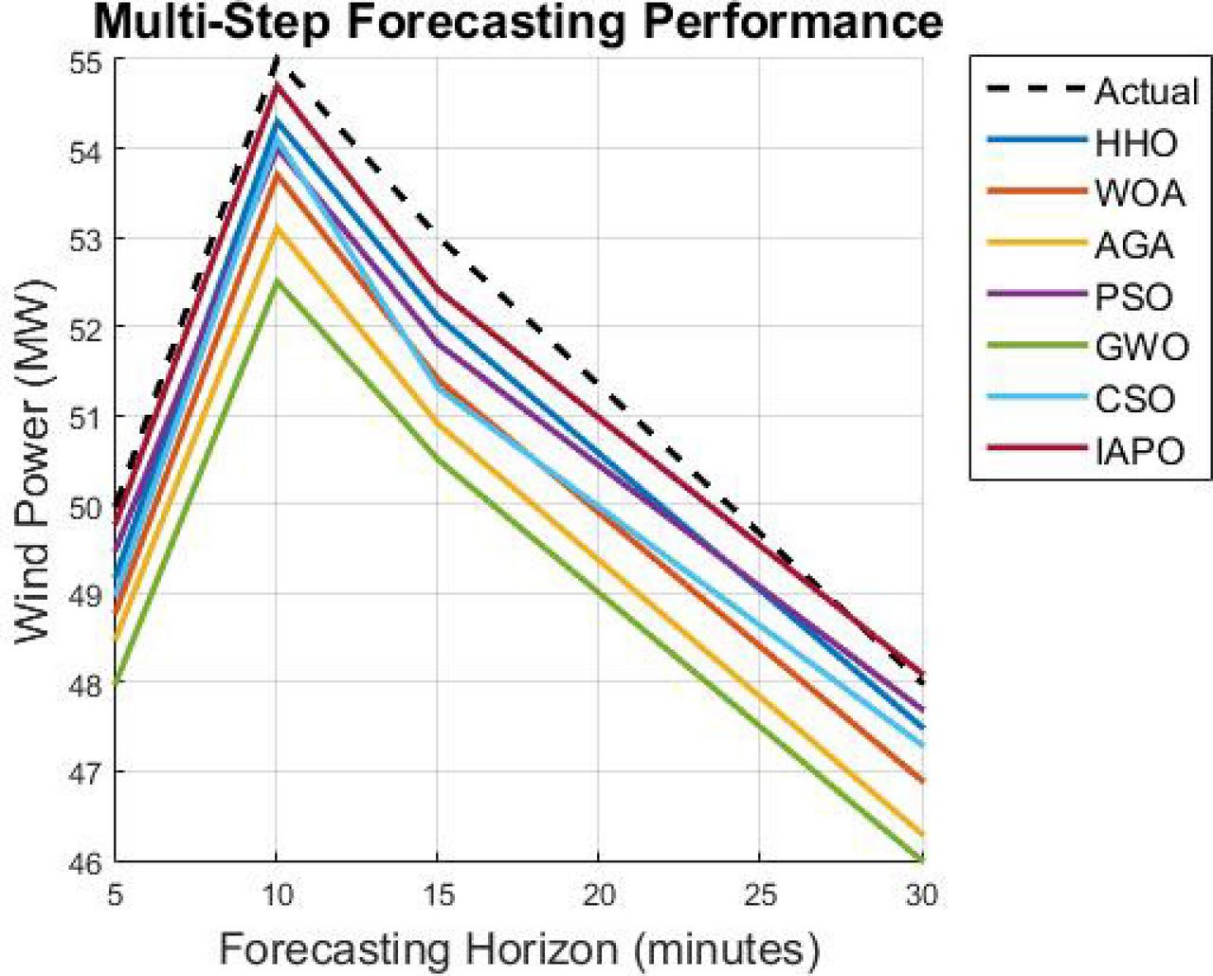
Forecasted wind power for each model on the WIND dataset.

**Fig. 7:**
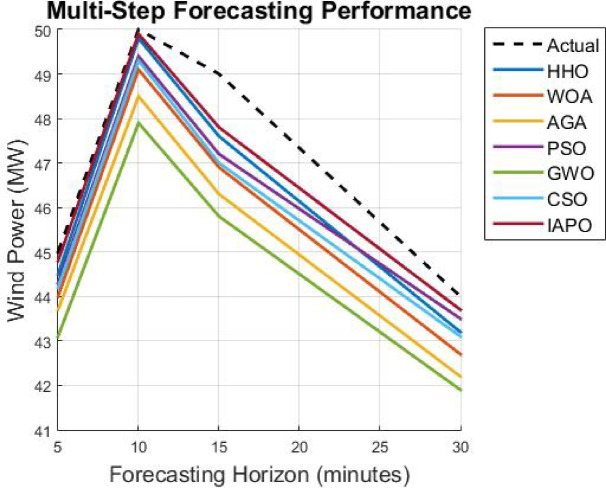
Forecasted wind power for each model on the EMD Wind dataset.

**Fig. 8:**
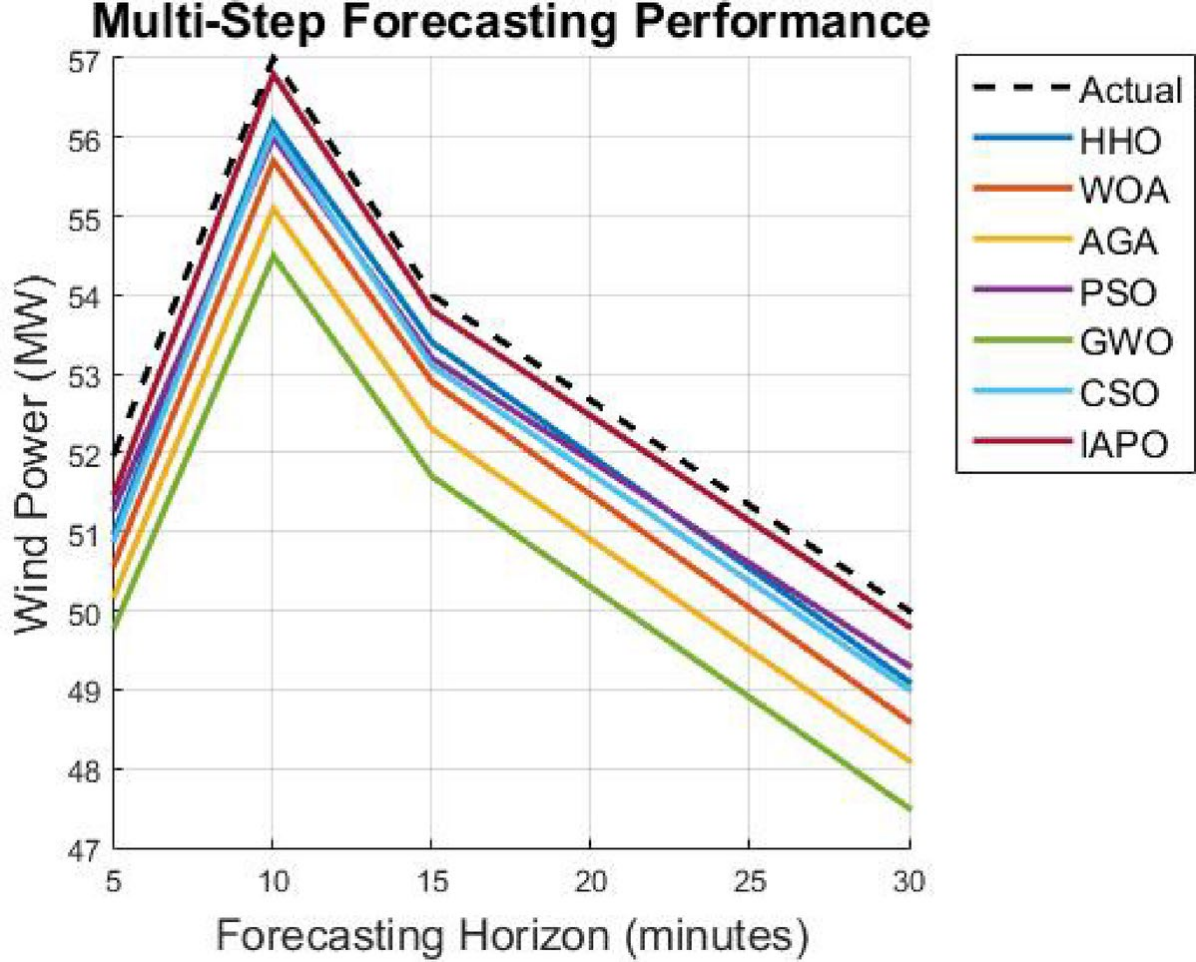
Forecasted wind power for each model on the WWSIS dataset.

**Fig. 9:**
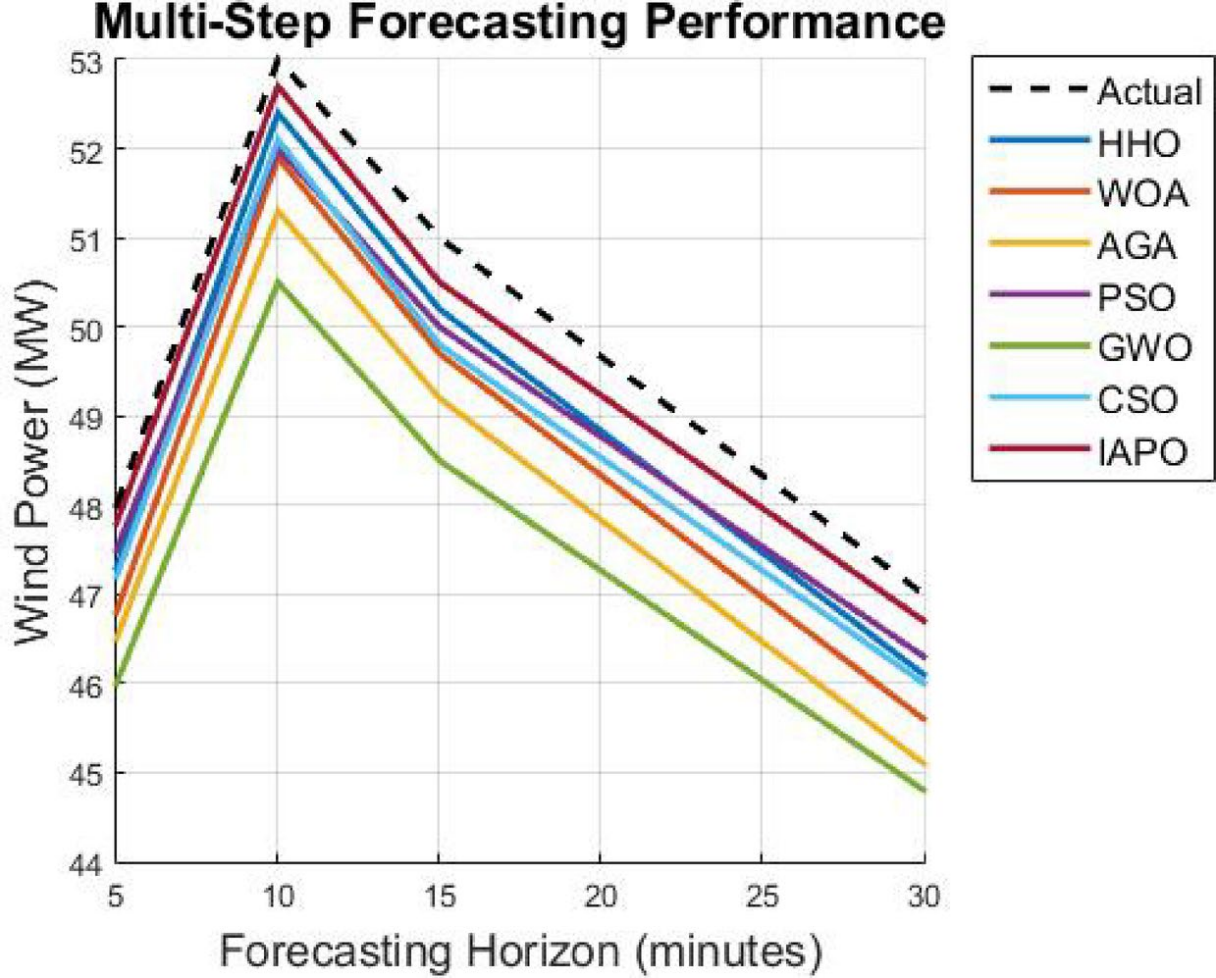
Forecasted wind power for each model on ERCOT Grid dataset.

Within Figs. 6, 7, 8 and 9, several revisions of the cross-sectional wind energy forecasting accuracy are demonstrated over four different data sources, namely NREL WIND, EMD Wind, WWSIS, and the ERCOT Grid. Each figure depicts the actual wind power values (ground truth) along with predictions made by HHO-ResNet, WOA-GRU, AGA-CNN, PSO-SVM, BiLSTM-GWO, CSO-CNN, and the IAPO-LSTM model. This comparison is done over the forecasting horizons of 5–10–15–30 min, indicating the model’s versatility in very short-term wind power forecasting.

Utilizing all datasets, IAPO-LSTM has shown the highest predictive accuracy by tracking the actual wind power values. This is especially evident in shorter forecasting horizons (5–10 min), where the proposed model effectively serves its purpose without significant fluctuations from the ground truth. The model IAPO-LSTM performs well due to its deep learning hybrid design with CNN for feature extraction and GRU for sequential learning, combined with dynamic hyper-parameter and feature selection through Artificial Protozoa Optimizer (APO). Together with the improvements made, these optimizations have IAPO-LSTM outperformed the other models as the error accumulation minimizes over multiple forecasting steps.

In comparison, other models seem to have increased forecasting mistakes as the prediction scope further goes to 30 min. More sophisticated traditional approaches like PSO-SVM and BiLSTM-GWO demonstrate greater error with respect to the actual wind power values at longer horizons. These results indicate that these approaches do not perform well in capturing non-linear temporal dependencies, which ultimately adds up the error. IAPO-LSTM captures most of these problems. Deep learning approaches such as HHO-ResNet and AGA-CNN captures the essence of the problem relatively well but still lag behind the accuracy of IAPO-LSTM. Deep learning models’ mistakes become more evident as forecasting ranges get deeper, further showing how poor they are in long-range forecasting.

A feature of the power datasets is that the overall performance of the wind power forecasting seems to depend on the local weather conditions as well as the features of the dataset itself. As an example, in Fig. 6 (NREL WIND), all models achieve relatively good accuracy at 5 min. Still, as the duration increases to more than 5 min, then we see that there is a deviation from the mean. The EMD Wind dataset shown in Fig. 7 demonstrates a similar pattern where IAPO-LSTM performs the best over all the methods regardless of the time interval. On the other hand, Figs. 8 and 9 (WWSIS and ERCOT Grid datasets) are perhaps much more difficult to forecast owing to the greater range in the wind power patterns. Regardless, IAPO-LSTM shows optimum performance during all the prediction intervals, while the other models suffer from large and volatile prediction errors.

These results strengthen the previously achieved outcomes associated with the IAPO-LSTM model’s forecasting abilities for very short-term wind power generation. The spatial and temporal learning components that are integrated into this model in the form of CNN for feature extraction, GRU for time series learning, and APO-AERM for optimization demonstrate that this model outperforms both deep learning and machine-learning-based models. This attribute, in addition to a range of high accuracy scores across multiple forecasting horizons, stretches its capabilities for real-time energy management alongside grid stability. The increasing forecasting horizon reveals greater discrepancies in the performance of the IAPO-LSTM models compared to the remaining models, which confirms its superior performance in multi-step forecasting problems as the model accumulates fewer and fewer cumulative errors.

**Fig. 10:**
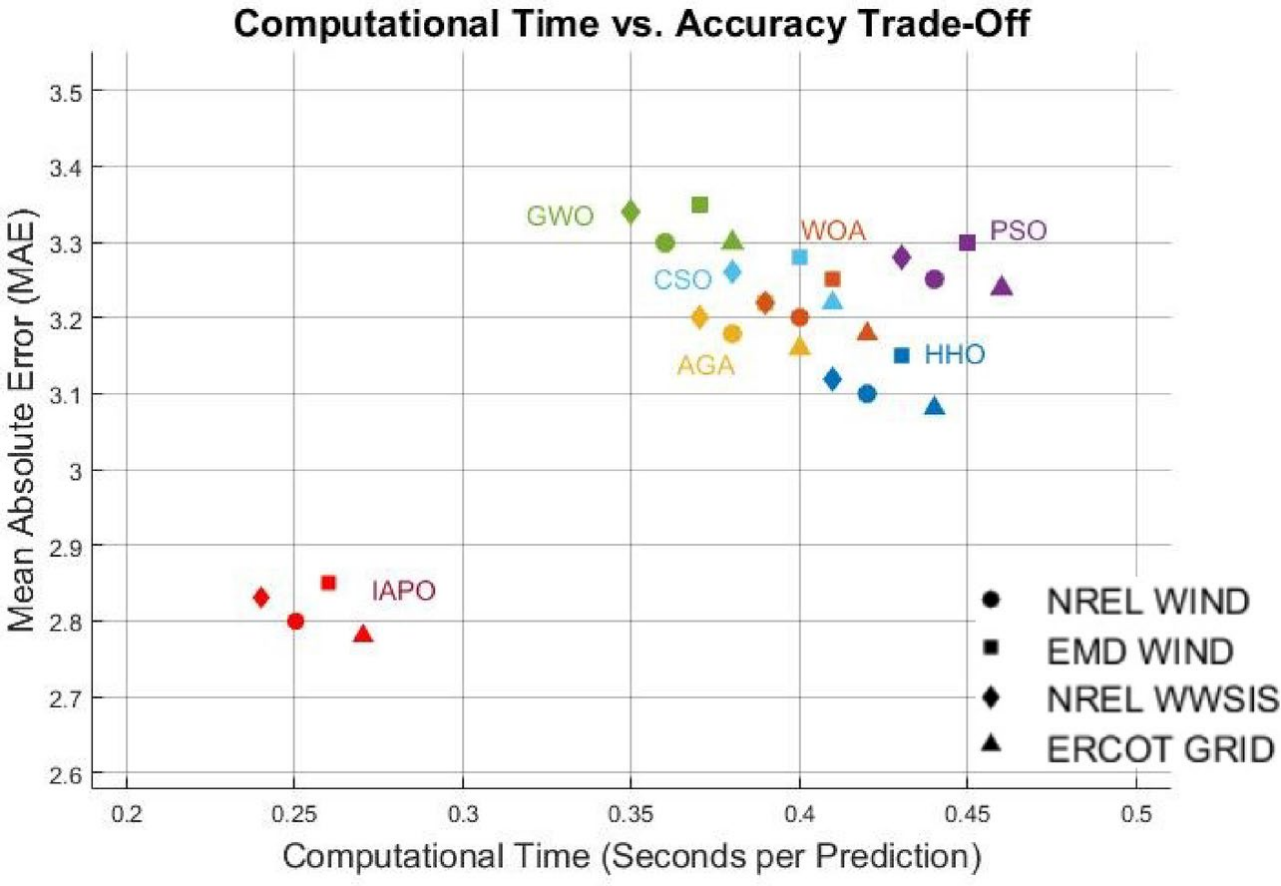
Scatter plot for all the comparative methods.

As illustrated in Fig. 10, the forecast accuracy and model effectiveness for each of the four wind power datasets, NREL WIND, EMD Wind, WWSIS, and ERCOT Grid, are compared. The datasets mentioned above are located on the x-axis of the Theil Inequality Coefficient (TIC), which measures the precision of the predictions made by the system. At the same time, mean absolute error (MAE), which measures accuracy, is placed on the y-axis. The size of the bubbles indicates the TIC. When the bubble is bigger, it shows the model is less accurate, and vice versa.

Both the accuracy range and effectiveness differ across the forecasting models and the results show a clear balance between the two factors. While deep-learning models such as HHO-ResNet and AGA-CNN are more accurate, they suffer from a moderate computational overhead. Traditional models tend to excel in time consumption but perform poorly when it comes to the biography. BiLSTM-GWO And PSO-SVM are the two outperforming models for the deep-learning category. Considering all CNN-based models, CSO-CNN and WOA-GRU experience moderate computational lag but do outperform the IAPO-LSTM model.

The most effective and precise of the forecasting approaches, The IAPO-LSTM model, marked in red performs significantly better than the rest with respect to accuracy and efficiency. It has the lowest MAE in all datasets and the least computational time. The incorporation of the Artificial Protozoa Optimizer (APO) integrated with an Adaptive Environmental Response Mechanism (AERM) alters hyperparameters, and feature selection in a non-linear manner increases efficiency and reduces wasted effort. Unlike other models, IAPO-LSTM strikes a unique balance of exploration and exploitation leading to the avoidance of local optima while guaranteeing fast convergence to the global optima.

Performance-wise, the ERCOT Grid dataset poses the highest difficulty in computation where all the models show slightly worse MAE alongside higher computational times. This is due to the higher difficulty and degeneracy of the wind power patterns in this dataset. Nevertheless, even here IAPO-LSTM achieves superior performance metrics in terms of MAE and execution time when compared to other models.

IAPO-LSTMs’ state-of-the-art methods outperform the rest with lower forecasting errors and minimal effort in Computation. It is achievable because IAPO-LSTM boasts greater accuracy and performs complex computations with unparalleled efficiency which is demonstrated in Fig. 10. This proves the IAPO-LSTM instruments in real-time wind power forecasting are a highly efficient solution for managing the energy grid. They are undoubtedly more useful for short-term forecasting.

**Fig. 11:**
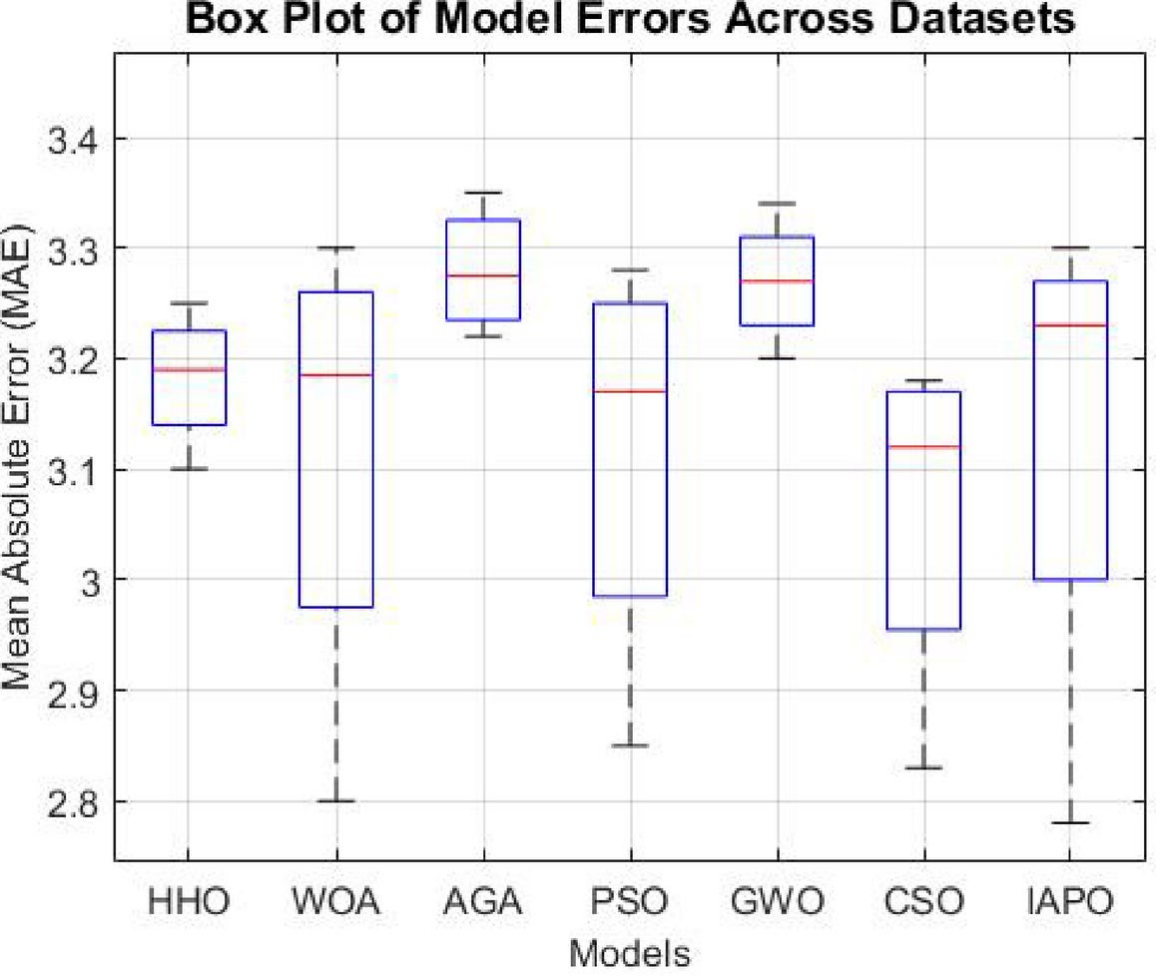
Boxplot error values for each model across all datasets.

The box plot in Fig. 11 graphically summarizes the MAE for the various models and compares their performance against four datasets, namely WIND from NREL, EMD WIND, NREL WWSIS, and the ERCOT Grid. The plot’s box feature, in particular, accounts for the variability of the prediction methods in the datasets, statistically portraying their accuracy.

As the results demonstrate, IAPO-LSTM, the model proposed, has the lowest MAE values for all datasets, being that the median remains lower IQRs across all datasets. These results point out that IAPO-LSTM provides better forecasts and exhibits less dispersion across varying forecasting scenarios, indicating that this model is the best approach for weather-associated electric power generation for wind power. The design of the model is capable of dynamic learning with meteorological stimuli due to the hyperparameter’s powerful optimization brought by the incorporation of APO and AERM.

Models such as PSO-SVM, BiLSTM-GWO, and CSO-CNN have, comparatively, greater median MAE values and wider IQR, which means less performance consistency. Considering these models have higher errors in prediction, the prediction accuracy fluctuation across datasets is more, and therefore makes them more unreliable in real-time predictions. While HHO-ResNet and AGA-CNN have outperformed some of the traditional ones, they still lag behind IAPO-LSTM in terms of accuracy and consistency.

The presence of outliers in some models further highlights their inconsistencies against varying windpower datasets. Models like WOA-GRU and BiLSTM-GWO do show a few extreme error outliers, which suggest a struggle with some particular complex forecasting problems. This is an important issue in short-term wind power prediction, where these models would be making real-time decisions with very unstable forecasts.

From the results, it is clear that the IAPO-LSTM model outperforms any other models in Fig. 11. IAPO-LSTM is the most attractive solution to renew the increasing concerns about grid stability and improve the reliability of forecasting renewables because deep learning (CNN-GRU) in conjunction with intelligent optimization (APO-AERM) guarantees a very low level of forecast errors while proving high accuracy.

**Fig. 12:**
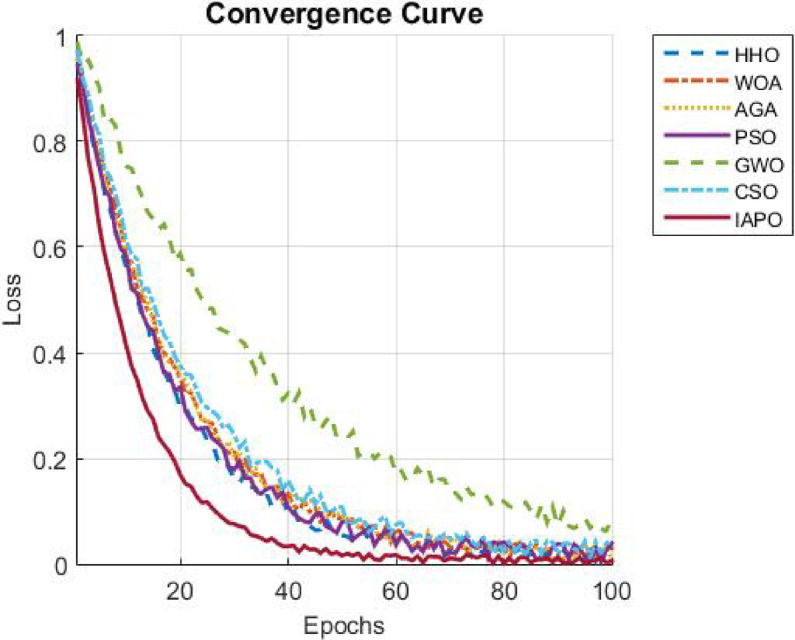
Plot convergence curves for each method on the WIND dataset.

**Fig. 13:**
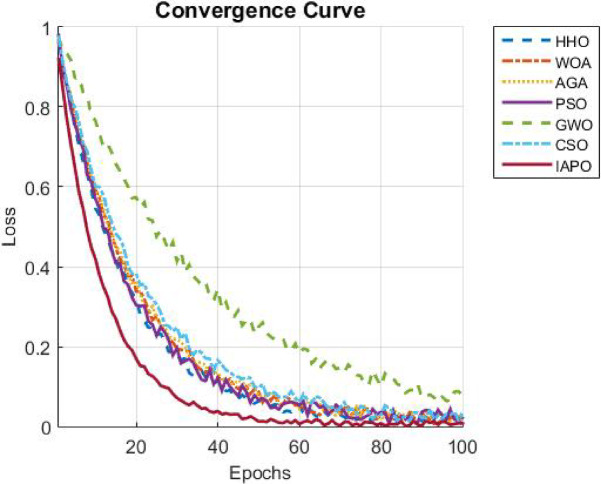
Plot convergence curves for each method on the EMD Wind dataset.

**Fig. 14:**
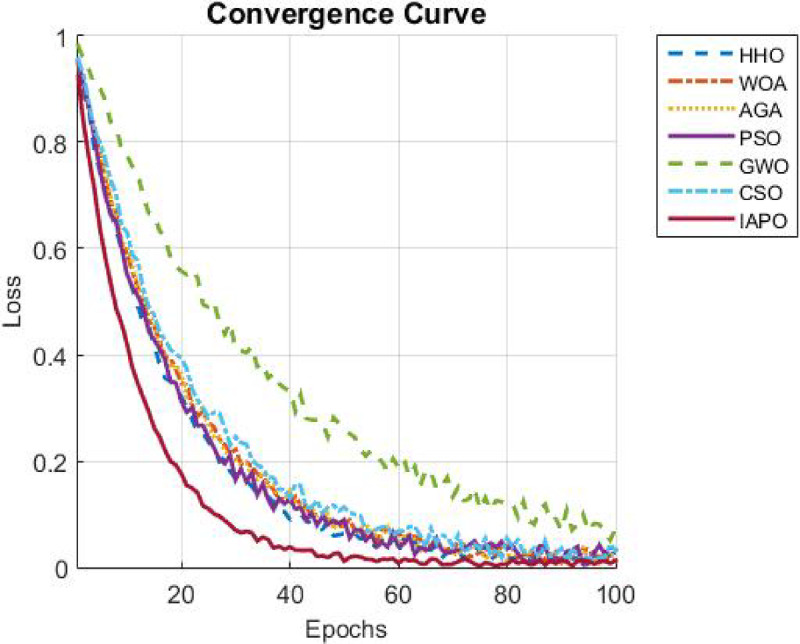
Plot convergence curves for each method on the WWSIS dataset.

**Fig. 15:**
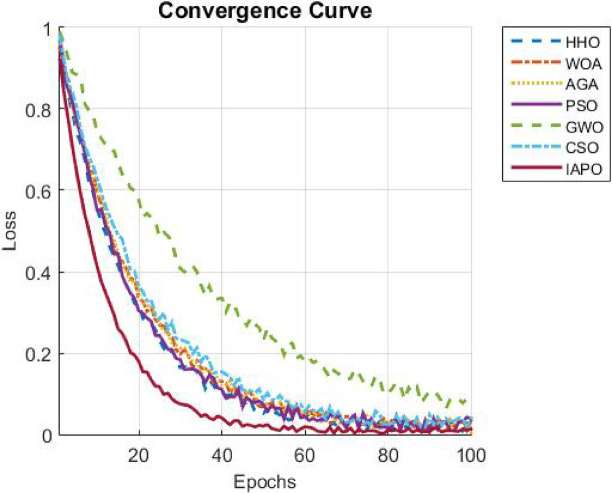
Plot convergence curves for each method on the ERCOT Grid dataset.

In the convergence analysis of the models for each of the datasets in WIND, EMD Wind, WWSIS, and ERCOT Grid, Figs. 12, 13, 14 and 15 show how each of the models has performed while training regarding the pace with which the model comes to minimizing the loss function throughout 100 epochs. These datasets were selected to make sure that the proposed method of multi-wind power forecasting checking was as effective and consistent as possible during induction and training procedures, as well as to check the method’s versatility regarding different power forecasts in different areas.

Perhaps the most important piece of information one can extract from these curves is that the proposed IAPO-LSTM model is the most effective when it comes to learning how to perform in forecasting, regardless of the scenario. The IAPO-LSTM model not only achieves better performance at lower values of loss than all other models but does so at an astonishing ower and recovery rate, thereby putting as practically no strain on the IAPO-LSTM model. This is helpful because loss minimization is the goal, and with IAPO-LSTM, it can be achieved at optimal conditions. The reason for this speed in loss function is the new Artificial Protozoa Optimizer (APO), which employs an Adaptive Environmental Response Mechanism (AERM) that makes augmenting hyperparameters and shifting between exploitation and exploration more efficient and responsive, thus enhancing the gradient updates. Loss curve fluctuations are decreased again, providing evidence of the model’s strength and stability.

Conversely, other models, including PSO-SVM and BiLSTM-GWO, have slower rates of convergence while experiencing higher loss retention over multiple epochs. This indicates that these approaches are not able to optimize their parameters fully and, hence, do not train efficiently. Models like CSO-CNN and AGA-CNN also display loss convergence but significant loss oscillations during training. This phenomenon stems from the models struggling with the adaptive learning of the non-stationary structures of wind power, which results in poor generalizing ability.

An additional notable remark is that WOA-GRU and HHO-ResNet, although performing better than other models, still show worse results when benchmarked against IAPO-LSTM regarding the convergence period. These models have longer convergence periods for optimal loss values, which suggests that the optimization process is more intricate. Poor responsiveness to the changes in the training datasets may compromise the utility of these models for real-time forecasting.

Also, in Figs. 12, 13, 14 and 15, the lead patterns that can be identified in performance metrics corroborate that IAPO-LSTM achieves training with significantly fewer epochs and achieves substantially lower loss metrics relative to the competition. This is valuable in real-world energy systems, where resources and time are crucial for the effective and timely forecast of wind energy. The integration of CNN for feature extraction, GRU for sequential learning, and APO-AERM for optimization bestows upon the proposed model a remarkable combination that is beneficial for both prediction and training accuracy.

**Fig. 16:**
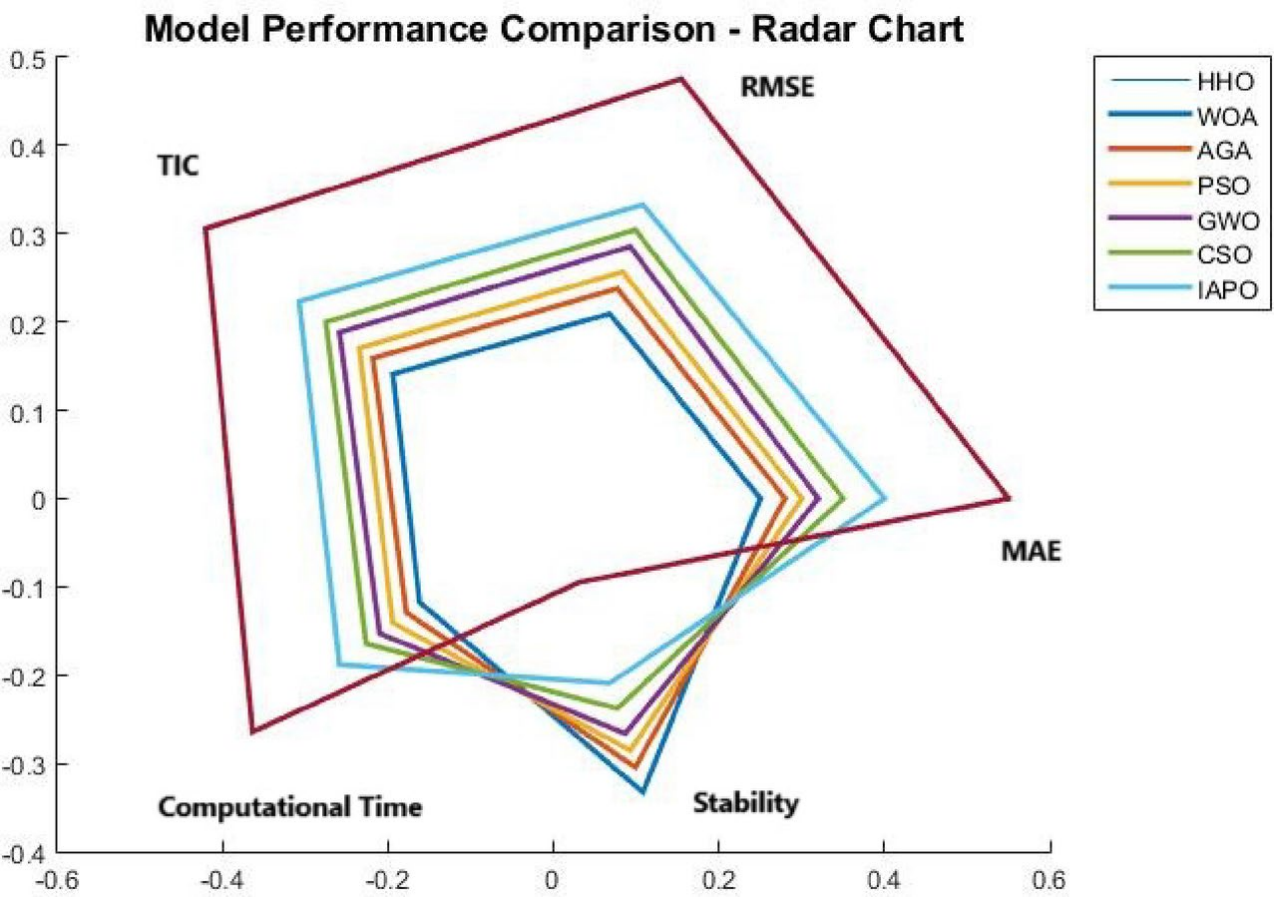
Radar chart.

The radar chart in Fig. 16 depicts the comparison of model performances based on the evaluation exercise of five selected wind power forecasting models with consideration to the following measures: Mean Absolute Error (MAE), Root Mean Square Error (RMSE), Theil’s Inequality Coefficient(TIC), Computation Time and Stability. This graphical illustration allows the performance of each forecasting model to be viewed in a manner that offers a consolidated measure of effectiveness and efficiency in a singular representation.

The critical point to note from the radar chart presented is the outperforming position among the rest of the models taken into consideration, in which the IAPO-LSTM model outstands its competitors due to a more optimal balance shown across every single evaluation metric. The proposed method has achieved the greatest reduction of MAE, RMSE, and TIC, suggesting the highest accuracy for very short-term wind power forecasting. Furthermore, IAPO-LSTM is also reported to have the lowest computational time during the tests, which supports the model efficiency claims. The IAPO-LSTM model also sustains a superior high stability result means that the model is able to retain consistent performance with low prediction accuracy variances across diverse datasets.

In contrast, the models BiLSTM-GWO, PSO-SVM, and AGA-CNN struggle with computational efficiency and effectiveness, which shows that these models’ methodology achieves adequate forecasting accuracy but has extensive resource bottom for their training and inference. This balance might restrict their use in real-time energy grid operations as they are time-sensitive. The CSO-CNN and WOA-GRU have moderate accuracy and lower stability, suggesting that their results are affected by the change of the training data and, therefore, can be questionable for practical application.

From the radar chart in Fig. 16, another key point is the performance of HHO-ResNet and WOA-GRU, which seems to be off the point of the radar’s accuracy range. TIC score optimization is sought through the other metrics, as with these models, the efficiency precision ratio does seem to provide sufficient reasoning in regard to their undue high-efficiency demands. These models might provide reasonable features in real-time forecasting scenarios on high-capacity wind power grids, but their high computation demands might render them obsolete.

Last but not least, the radar chart supports the claim that the integration of the Artificial Protozoa Optimizer with the AERM system within the IAPO-LSTM model is beneficial. The ability of APO to hyperparameter tune while dynamically maintaining an optimal exploration-exploitation balance aids in faster convergence and predictive accuracy. These results also help confirm that IAPO-LSTM is indeed the most accurate and, at the same time, the most stable and efficient computationally, making it the optimal choice for forecasting wind energy power in real-time as well as for energy management systems.

### Strengths, discussion, and limitations

The proposed IAPO-LSTM framework has shown great performance in very short-term wind power estimation with the help of extensive tests performed over several datasets. The combination of Artificial Protozoa Optimiser (APO) and Adaptive Environmental Response Mechanism (AERM) is equally proficient in dynamic hyperparameter optimizing, predictive accuracy, and computational resources. Function class feature extraction from CNN, together with the GRU-driven temporal learning guarantees the best capturing of all spatial and sequential dependencies of the wind power data. Across all the datasets, lower forecasting errors (MAE, RMSE, TIC) and much less computation time were achieved with IAPO-LSTM compared to the other methods, further making IAPO-LSTM an ideal forecasting model for energy management systems.

Effective execution of the method proposed in this paper is attributed to one of its greatest strengths, which is the ability to maintain accuracy and efficiency for a wide variety of forecasting horizons (5, 10, 15, and 30 min). This feature enables the model to work in ‘real-time’ contexts, such as during power grid control processes. Furthermore, the automatic feature used in the model is capable of meteorologically developing bone FIS devoid of obesity features. This aids in ensuring that only the most relevant variables are used in the forecasting. This obviates and enhances the logic of the model and, at the same time, increases model fit computation, which results in the elimination of large-scale inefficiency for wind energy foreseeing processes.

Nevertheless, some shortcomings still exist, especially when comparing new approaches with existing approaches based on deep learning and even hybrid models. Specifically, a noted limitation that stands out is the computation during the training phase in regard to complex models. Despite this challenge, it is expected the APO will increase the speed of convergence throughout the model. As is the case with many heuristic approaches, while APO improves the speed of convergence compared to traditional techniques, it does so at the cost of requiring many cycles during the initialization stage to set a reinforcement learning parameter, which is not expected accurately. Thus, while the inference appeal makes use of a low-dimensional dataset to avoid long training times, the optimal setting is severe computational spending during the cost.

Another issue to consider is the model’s response to extreme weather conditions. Although the process of APO-driven heuristics allows for some flexibility with respect to different weather conditions, any abrupt variations in the atmosphere, such as storms or quick changes in wind direction, tend to mitigate the accuracy of the predictions made by the model. Solving this issue might require more work on the developed feature extraction techniques or incorporating more information like satellite images or local deviations of air pressure.

In the coming years, the implementation of IAPO-LSTM can be improved in many ways. The first direction is through integrating meta-learning into APO, as this may improve a model’s ability to adapt and perform flexibly in more complex environments. The second direction is more focused on the data level. It suggests the use of data fusion technologies for the incorporation of satellite wind measurements, turbine sensors, and microwave hypersonic weather models. Finally, tests on the model, and consequently the grid as well, could be done in real-time where wind energy data is actively streamed. This would test the model’s practicality and its functioning under operational conditions.

The IAPO-LSTM framework is a notable step forward in very short-term wind power forecasting. The deep learning and bio-inspired optimization, along with adaptive exploration mechanisms, guarantees accuracy while being computationally efficient and practical. Although there are some unresolved issues, the results indicate that IAPO-LSTM can significantly contribute toward real-time energy forecasting, smart grid optimization, and renewable energy utilization.

## Conclusion and future work

Power grid operators face unique challenges in integrating renewable sources into the grid, with forecasting wind power usage remaining one of the most difficult ones. The volatility of wind speeds, along with the complex and changing multi-measurement influences, require advanced, flexible, and scalable forecasting models. The study presents IAPO-LSTM, a novel hybrid deep learning framework that integrates features from Convolutional Neural Networks (CNNs), Gated Recurrent Units (GRUs), and an Artificial Protozoa Optimizer (APO) enhanced by the AERM for deep hyperparameter optimization. The combination of APO with AERM allows controlling the “intelligent” optimization through adaptive exploration and exploitation processes, which leads to increased accuracy and efficiency and greater model dependence stability. After exhaustive testing on four real-life wind power datasets: NREL WIND, EMD WIND, WWSIS, and ERCOT GRID, the results showed that IAPO-LSTM trained on these datasets outperforms six other models that claim to be the best in the field, with higher forecasting efficiency and lower forecast errors (MAE, RMSE, TIC), as well as reduced computation time and better model accuracy over various forecasting periods.

Among the noteworthy components highlighted in the study is the ability of the model to provide high-level feature interpretability as well as operational efficiency stemming from the removal of unnecessary meteorological features, which utilizes a great deal of computation with high accuracy of prediction. The results from multi-horizon forecasting showed that the IAPO-LSTM model performs satisfactorily across different short-term time frame windows of 5, 10, 15, and 30 min, suggesting its applicability for real-time grid applications. The analysis suggests that conventional approaches have inadequacies like PSO, WOA, and GWO local optima problems. At the same time, AOP-AERM is able to change its search step parameters to eliminate convergence stagnation. In addition, the statistical tests performed also confirmed the results of IAPO-LSTM being the most robust solution across all datasets, further substantiating the theory of the model being a good solution for estimating time series data. Nonetheless, the study also recognized some of the weaknesses that range from the focus on high computational overhead together with low training performance, which advanced optimization algorithms are required to make improvements to the applicability in practice.

Many developments can be made in the practical utilization of IAPO-LSTM, as well as its performance capabilities. For example, increasing the dataset to include additional meteorological factors, such as turbulence intensity, cloud cover, and atmospheric stability, should allow for adaptation to be greater. Furthermore, satellite-based wind flow analysis and sensor-based turbine readings should improve the robustness of the forecast for extreme weather scenarios. Moreover, integrating APO with reinforcement learning-based meta-optimization should allow the model to modify hyperparameters based on real-time forecasting performance. Finally, the practical application of the model and its efficiency, along with the issues of scalability, can be examined in greater depth by deploying the IAPO-LSTM framework in live smart grid environments and working with stakeholders in the energy sector.

In addition, future research will need to consider energy-efficient machine learning models that enhance forecast accuracy while reducing energy expenditure during model training and inference. This would further AI-enabled forecasting solutions. Another area of research is expanding the forecasting horizon to include medium and long-term periods while still maintaining accuracy, which would assist power system operators with long-term strategic planning and stability of the grid. Finally, implementing an uncertainty quantification mechanism into IAPO-LSTM could enable the model to construct prediction intervals, thereby providing energy managers with probabilistic information about the level of confidence in the forecast.

Lastly, the energy sector is always in need of optimization, and with innovative breakthroughs in sizable areas such as deep learning integrated with biology, the IAPO-LSTM framework stands as a paradigm shift in wind power forecasting. This multidisciplinary strategy is able to provide high degrees of automation, reliability, and efficiency to renewable energy systems by hyper-adaptive powers. These revolutionary boundaries set encourage AI systems embedded with smart, advanced analyzing tools, paving the pathway for adapting robust AI-powered forecasting systems in the smart energy world.

## Data Availability

Data is available from the Laith Abualigah upon reasonable request.

## References

[1] Bazmi, A. A. & Zahedi, G. Sustainable energy systems: role of optimization modeling techniques in power generation and supply—A review. *Renew. Sustain. Energy Rev.* **15**, 3480–3500 (2011).

[2] Hassan, Q. et al. The renewable energy role in the global energy transformations. *Renew. Energy Focus*. **48**, 100545 (2024).

[3] Singla, P., Saroha, S., Duhan, M., Shekher, V. & Singh, K. A solar irradiance forecasting model using iterative filtering and bidirectional long short-term memory. *Energy Sour. Part A Recover. Utilization Environ. Eff.* **46**, 8202–8222 (2024).

[4] Blaga, R. et al. A current perspective on the accuracy of incoming solar energy forecasting. *Prog. Energy Combust. Sci.* **70**, 119–144 (2019).

[5] Zendehboudi, A., Baseer, M. A. & Saidur, R. Application of support vector machine models for forecasting solar and wind energy resources: A review. *J. Clean. Prod.* **199**, 272–285 (2018).

[6] Singla, P., Duhan, M. & Saroha, S. A point and interval forecasting of solar irradiance using different decomposition based hybrid models. *Earth Sci. Inf.* **16**, 2223–2240 (2023).

[7] Alkhayat, G. & Mehmood, R. A review and taxonomy of wind and solar energy forecasting methods based on deep learning. *Energy AI*. **4**, 100060 (2021).

[8] Aslam, S. et al. A survey on deep learning methods for power load and renewable energy forecasting in smart microgrids. *Renew. Sustain. Energy Rev.* **144**, 110992 (2021).

[9] Vlahogianni, E. I. Optimization of traffic forecasting: intelligent surrogate modeling. *Transp. Res. Part. C: Emerg. Technol.* **55**, 14–23 (2015).

[10] Tao, Q., Liu, F., Li, Y. & Sidorov, D. Air pollution forecasting using a deep learning model based on 1D Convnets and bidirectional GRU. *IEEE Access.* **7**, 76690–76698 (2019).

[11] Dincer, F. The analysis on wind energy electricity generation status, potential and policies in the world. *Renew. Sustain. Energy Rev.* **15**, 5135–5142 (2011).

[12] Kaplan, Y. A. Overview of wind energy in the world and assessment of current wind energy policies in Turkey. *Renew. Sustain. Energy Rev.* **43**, 562–568 (2015).

[13] Liu, L. et al. Ultra-short-term wind power forecasting based on deep bayesian model with uncertainty. *Renew. Energy*. **205**, 598–607 (2023).

[14] Wang, J., Hu, J. & Ma, K. Wind speed probability distribution Estimation and wind energy assessment. *Renew. Sustain. Energy Rev.* **60**, 881–899 (2016).

[15] Liu, H. & Chen, C. Data processing strategies in wind energy forecasting models and applications: A comprehensive review. *Appl. Energy*. **249**, 392–408 (2019).

[16] Wahdany, D., Schmitt, C. & Cremer, J. L. More than accuracy: end-to-end wind power forecasting that optimises the energy system. *Electr. Power Syst. Res.* **221**, 109384 (2023).

[17] Kaur, G. & Sharma, A. A deep learning-based model using hybrid feature extraction approach for consumer sentiment analysis. *J. Big Data*. **10**, 5 (2023). 36686621 10.1186/s40537-022-00680-6 PMC9838421

[18] Dargan, S., Kumar, M., Ayyagari, M. R. & Kumar, G. A survey of deep learning and its applications: a new paradigm to machine learning. *Arch. Comput. Methods Eng.* **27**, 1071–1092 (2020).

[19] Kachare, P. et al. LCADNet: a novel light CNN architecture for EEG-based Alzheimer disease detection. *Phys. Eng. Sci. Med.* 1–14 (2024). 10.1007/s13246-024-01425-w 38862778

[20] Li, W. et al. Prediction of dissolved oxygen in a fishery pond based on gated recurrent unit (GRU). *Inform. Process. Agric.* **8**, 185–193 (2021).

[21] Sajjad, M. et al. A novel CNN-GRU-based hybrid approach for short-term residential load forecasting. *Ieee Access.* **8**, 143759–143768 (2020).

[22] ArunKumar, K., Kalaga, D. V., Kumar, C. M. S., Kawaji, M. & Brenza, T. M. Forecasting of COVID-19 using deep layer recurrent neural networks (RNNs) with gated recurrent units (GRUs) and long short-term memory (LSTM) cells. *Chaos Solitons Fractals*. **146**, 110861 (2021). 33746373 10.1016/j.chaos.2021.110861 PMC7955925

[23] Zhang, J., Cheng, C. & Yu, S. Recognizing the mapping relationship between wind power output and meteorological information at a Province level by coupling GIS and CNN technologies. *Appl. Energy*. **360**, 122791 (2024).

[24] Yu, G. et al. Short term wind power prediction for regional wind farms based on spatial-temporal characteristic distribution. *Renew. Energy*. **199**, 599–612 (2022).

[25] Kisvari, A., Lin, Z. & Liu, X. Wind power forecasting–A data-driven method along with gated recurrent neural network. *Renew. Energy*. **163**, 1895–1909 (2021).

[26] Boucetta, L. N., Amrane, Y. & Arezki, S. Wind power forecasting using a GRU attention model for efficient energy management systems. *Electr. Eng.*, pp. 1–26, (2024).

[27] Liao, L., Li, H., Shang, W. & Ma, L. An empirical study of the impact of hyperparameter tuning and model optimization on the performance properties of deep neural networks. *ACM Trans. Softw. Eng. Methodol. (TOSEM)*. **31**, 1–40 (2022).

[28] Nematzadeh, S., Kiani, F., Torkamanian-Afshar, M. & Aydin, N. Tuning hyperparameters of machine learning algorithms and deep neural networks using metaheuristics: A bioinformatics study on biomedical and biological cases. *Comput. Biol. Chem.* **97**, 107619 (2022). 35033837 10.1016/j.compbiolchem.2021.107619

[29] Lim, J., Lim, J., Baskaran, V. M. & Wang, X. A deep context learning based PCB defect detection model with anomalous trend alarming system. *Results Eng.* **17**, 100968 (2023).

[30] Onufriev, A. V. & Izadi, S. Water models for biomolecular simulations. *Wiley Interdisciplinary Reviews: Comput. Mol. Sci.* **8**, e1347 (2018).

[31] Varshney, M., Kumar, P. & Abualigah, L. Hybridizing Remora and Aquila optimizer with dynamic oppositional learning for structural engineering design problems. *J. Comput. Appl. Math.* **462**, 116475 (2025).

[32] Biswas, S. et al. Integrating differential evolution into gazelle optimization for advanced global optimization and engineering applications. *Comput. Methods Appl. Mech. Eng.* **434**, 117588 (2025).

[33] Ghasemi, M. et al. An efficient bio-inspired algorithm based on humpback whale migration for constrained engineering optimization. *Results Eng.* 104215 (2025).

[34] Abualigah, L. et al. ,*., Adaptive Gbest-Guided Atom Search Optimization for Designing Stable Digital IIR Filters, Circuits, Systems, and Signal Processing, pp. 1–23*, (2025).

[35] Wu, J. et al. Hyperparameter optimization for machine learning models based on bayesian optimization. *J. Electron. Sci. Technol.* **17**, 26–40 (2019).

[36] Hartley, R. I. & Kahl, F. Global optimization through rotation space search. *Int. J. Comput. Vision*. **82**, 64–79 (2009).

[37] Rahmani, R., Yusof, R., Seyedmahmoudian, M. & Mekhilef, S. Hybrid technique of ant colony and particle swarm optimization for short term wind energy forecasting. *J. Wind Eng. Ind. Aerodyn.* **123**, 163–170 (2013).

[38] Ren, C. et al. Optimal parameters selection for BP neural network based on particle swarm optimization: A case study of wind speed forecasting. *Knowl. Based Syst.* **56**, 226–239 (2014).

[39] Chang, W. Y. An RBF neural network combined with OLS algorithm and genetic algorithm for Short-Term wind power forecasting. *J. Appl. Math.* **2013**, 971389 (2013).

[40] Shahid, F., Zameer, A. & Muneeb, M. A novel genetic LSTM model for wind power forecast. *Energy* **223**, 120069 (2021).

[41] Abualigah, L., Diabat, A., Mirjalili, S., Abd Elaziz, M. & Gandomi, A. H. The arithmetic optimization algorithm. *Comput. Methods Appl. Mech. Eng.* **376**, 113609 (2021).

[42] Hou, G., Wang, J. & Fan, Y. Multistep short-term wind power forecasting model based on secondary decomposition, the kernel principal component analysis, an enhanced arithmetic optimization algorithm, and error correction. *Energy* **286**, 129640 (2024).

[43] Al-Ibraheemi, Z. & Al-Janabi, S. Sustainable energy: advancing wind power forecasting with grey Wolf optimization and GRU models. *Results Eng.* **24**, 102930 (2024).

[44] Cai, Z. et al. Gray Wolf optimization-based wind power load mid-long term forecasting algorithm. *Comput. Electr. Eng.* **109**, 108769 (2023).

[45] Črepinšek, M., Liu, S. H. & Mernik, M. Exploration and exploitation in evolutionary algorithms: A survey. *ACM Comput. Surv. (CSUR)*. **45**, 1–33 (2013).

[46] Son, P. V. H. & Nguyen Dang, N. T. Solving large-scale discrete time–cost trade-off problem using hybrid multi-verse optimizer model. *Scientific Reports* **13**, 1987 (1987). 10.1038/s41598-023-29050-9 PMC9898292 36737486

[47] Ekinci, S., Izci, D., Abualigah, L. & Zitar, R. A. A modified oppositional chaotic local search strategy based Aquila optimizer to design an effective controller for vehicle cruise control system. *J. Bionic Eng.* **20**, 1828–1851 (2023).

[48] Liu, H., Chen, C., Lv, X., Wu, X. & Liu, M. Deterministic wind energy forecasting: A review of intelligent predictors and auxiliary methods. *Energy. Conv. Manag.* **195**, 328–345 (2019).

[49] Yao, Z., Wang, Z., Wang, D., Wu, J. & Chen, L. An ensemble CNN-LSTM and GRU adaptive weighting model based improved sparrow search algorithm for predicting runoff using historical meteorological and runoff data as input. *J. Hydrol.* **625**, 129977 (2023).

[50] Torres, J. F., Hadjout, D., Sebaa, A., Martínez-Álvarez, F. & Troncoso, A. Deep learning for time series forecasting: a survey. *Big Data*. **9**, 3–21 (2021). 33275484 10.1089/big.2020.0159

[51] Xiao, M. et al. Addressing Overfitting Problem in Deep Learning-Based Solutions for Next Generation Data‐Driven Networks. *Wireless Communications and Mobile Computing* **2021**, 8493795 (2021).

[52] Abualigah, L. & Diabat, A. A comprehensive survey of the grasshopper optimization algorithm: results, variants, and applications. *Neural Comput. Appl.* **32**, 15533–15556 (2020).

[53] Masood, J. A. I. S. et al. A hybrid deep learning model to predict High-Risk students in virtual learning environments. *IEEE Access.* (2024).

[54] Umamaheswari, P. & Ramaswamy, V. An integrated framework for rainfall prediction and analysis using a stacked heterogeneous ensemble model (SHEM). *Expert Syst. Appl.* **256**, 124831 (2024).

[55] Richards, B. A. et al. A deep learning framework for neuroscience. *Nat. Neurosci.* **22**, 1761–1770 (2019). 31659335 10.1038/s41593-019-0520-2 PMC7115933

[56] Imrana, Y. et al. CNN-GRU-FF: a double-layer feature fusion-based network intrusion detection system using convolutional neural network and gated recurrent units. *Complex. Intell. Syst.* **10**(3), 3353–3370 (2024).

[57] Wang, K., Qi, X. & Liu, H. Photovoltaic power forecasting based LSTM-Convolutional Network. *Energy* **189**, 116225 (2019).

[58] Li, D. et al. A short-term electric load forecast method based on improved sequence-to-sequence GRU with adaptive Temporal dependence. *Int. J. Electr. Power Energy Syst.* **137**, 107627 (2022).

[59] Guo, X., Zhan, Y., Zheng, D., Li, L. & Qi, Q. Research on short-term forecasting method of photovoltaic power generation based on clustering SO-GRU method. *Energy Rep.* **9**, 786–793 (2023).

[60] Wang, X. et al. Artificial protozoa optimizer (APO): A novel bio-inspired metaheuristic algorithm for engineering optimization. *Knowl. Based Syst.* **295**, 111737 (2024).

[61] Antonanzas, J. et al. *Rev. Photovolt. Power Forecast. Solar Energy*, **136**, 78–111, (2016).

[62] Monteiro, C. et al. Wind power forecasting: state-of-the-art 2009, Argonne National Lab.(ANL), Argonne, IL (United States)2009.

[63] Kumar, K., Prabhakar, P., Verma, A., Saroha, S. & Singh, K. Advancements in wind power forecasting: A comprehensive review of artificial intelligence-based approaches. *Multimedia Tools Appl.*, pp. 1–30, (2024).

[64] Saroha, S., Zurek-Mortka, M., Szymanski, J. R., Shekher, V. & Singla, P. Forecasting of market clearing volume using wavelet packet-based neural networks with tracking signals. *Energies* **14**, 6065 (2021).

[65] Bashir, H., Sibtain, M., Hanay, Ö., Azam, M. I. & Saleem, S. Decomposition and Harris hawks optimized multivariate wind speed forecasting utilizing sequence2sequence-based spatiotemporal attention. *Energy* **278**, 127933 (2023).

[66] Lin, Z. Enhanced GRU-based regression analysis via a diverse strategies Whale optimization algorithm. *Sci. Rep.* **14**, 25629 (2024). 39465326 10.1038/s41598-024-77517-0 PMC11514269

[67] Fu, W. et al. A hybrid approach for multi-step wind speed forecasting based on two-layer decomposition, improved hybrid DE-HHO optimization and KELM. *Renew. Energy*. **164**, 211–229 (2021).

[68] Xie, Y., Li, C., Li, M., Liu, F. & Taukenova, M. An overview of deterministic and probabilistic forecasting methods of wind energy. *Iscience* **26**, (2023). 10.1016/j.isci.2022.105804 PMC9823194 36624842

[69] Li, B., Sun, F., Lian, Y., Xu, J. & Zhou, J. A variational mode Decomposition–Grey Wolf Optimizer–Gated recurrent unit model for forecasting water quality parameters. *Applied Sci.* **14**(14), 6111 (2024).

[70] Li, S. et al. Dconformer: A denoising convolutional transformer with joint learning strategy for intelligent diagnosis of bearing faults. *Mechanical Systems and Signal Processing* **210**, 111142 (2024).

[71] Yang, W., Sun, S., Hao, Y. & Wang, S. A novel machine learning-based electricity price forecasting model based on optimal model selection strategy. *Energy* **238**, 121989 (2022).

[72] Faruque, M. O., Hossain, M. A., Islam, M. R., Alam, S. M. & Karmaker, A. K. Very short-term wind power forecasting for real-time operation using hybrid deep learning model with optimization algorithm. *Clean. Energy Syst.* **9**, 100129 (2024).

